# Addressing the quantitative conversion bottleneck in single-atom catalysis

**DOI:** 10.1038/s41467-022-30551-w

**Published:** 2022-05-19

**Authors:** Zhongxin Chen, Jingting Song, Rongrong Zhang, Runlai Li, Qikun Hu, Pingping Wei, Shibo Xi, Xin Zhou, Phuc T. T. Nguyen, Hai M. Duong, Poh Seng Lee, Xiaoxu Zhao, Ming Joo Koh, Ning Yan, Kian Ping Loh

**Affiliations:** 1 Department of Chemistry, National University of Singapore, 3 Science Drive 3, Singapore, 117543 Singapore; 2 Joint School of NUS and TJU, International Campus of Tianjin University, Fuzhou, 350207 China; 3 College of Polymer Science & Engineering, Sichuan University, Chengdu, 610065 P. R. China; 4 Department of Chemical and Biomolecular Engineering, National University of Singapore, Singapore, 117585 Singapore; 5 Institute of Chemical and Engineering Sciences, Agency for Science, Technology and Research (A*STAR), 1 Pesek Road, Jurong Island, Singapore, 627833 Singapore; 6 Department of Mechanical Engineering, National University of Singapore, Singapore, 117575 Singapore; 7 School of Materials Science and Engineering, Peking University, Beijing, 100871 People’s Republic of China

**Keywords:** Heterogeneous catalysis, Two-dimensional materials, Chemical engineering

## Abstract

Single-atom catalysts (SACs) offer many advantages, such as atom economy and high chemoselectivity; however, their practical application in liquid-phase heterogeneous catalysis is hampered by the productivity bottleneck as well as catalyst leaching. Flow chemistry is a well-established method to increase the conversion rate of catalytic processes, however, SAC-catalysed flow chemistry in packed-bed type flow reactor is disadvantaged by low turnover number and poor stability. In this study, we demonstrate the use of fuel cell-type flow stacks enabled exceptionally high quantitative conversion in single atom-catalyzed reactions, as exemplified by the use of Pt SAC-on-MoS_2_/graphite felt catalysts incorporated in flow cell. A turnover frequency of approximately 8000 h^−1^ that corresponds to an aniline productivity of 5.8 g h^−1^ is achieved with a bench-top flow module (nominal reservoir volume of 1 cm^3^), with a Pt_1_-MoS_2_ catalyst loading of 1.5 g (3.2 mg of Pt). X-ray absorption fine structure spectroscopy combined with density functional theory calculations provide insights into stability and reactivity of single atom Pt supported in a pyramidal fashion on MoS_2_. Our study highlights the quantitative conversion bottleneck in SAC-mediated fine chemicals production can be overcome using flow chemistry.

## Abstract (web summary)

The practical application of single atom catalyst (SAC) in liquid-phase heterogeneous catalysis is hampered by the productivity bottleneck as well as catalyst leaching. Here, a bench-top, fast-flow reactor integrated with Pt1-MoS2 SAC was fabricated for continuous production of multifunctional anilines (28 examples) at a record productivity of 5.8 g h-1.

## Introduction

Single-atom catalysts (SACs) supported on heterogeneous substrates are often employed to emulate the coordination environment of homogeneous catalysts and the ease of separation of heterogeneous catalysts^1–3^. Moreover, SAC-mediated hydrogenation and oxidative reactions with good chemoselectivity have been demonstrated, including the late-stage functionalisation of pharmaceuticals^4–7^. However, most of these reactions are not scalable owing to their low quantitative conversion in batch reactors, which is fundamentally limited by the low mass loading of the SACs on supports. Beyond a threshold concentration, the aggregation of SAC occurs and this compromises the chemoselectivity unique to SAC^1^. Other pertinent issues include the insufficient activation of complex reactants by single metal sites and metal leaching from the supports in the solution. Thus, considerable efforts have been devoted to the understanding of catalysis at the atomic level and to design powerful and leaching-resistant SACs^8,9^.

From a practical perspective, the industrial adoption of SACs in liquid-phase transformations is hampered by insufficient productivity^1–3^. Flow chemistry can be applied to maximise the throughput of the reaction by promoting mass diffusion kinetics at the multiphasic interface and to minimise waste generation during catalyst separation^10–12^. However, commercial packed-bed reactors for powder catalysis require high operating pressures to attain a satisfactory flow rate^13,14^. Such high pressures may deactivate the SACs or lead to catalyst leaching, especially in the presence of external ligands in the solution^15^. Packed-bed reactors also require a large amount of catalyst, posing technical difficulties to replace the catalyst for screening purposes^13,14^. Moreover, complex temperature control is required to minimise the side reactions and the number of ineffective regions caused by the temperature gradient inside the reactor. Because of these reasons, only a few demonstrations of continuous-flow operations in SAC-mediated organic transformations have been reported. For example, the Pd_1_-catalysed Suzuki coupling reaction performed in a packed-bed flow reactor exhibited a low productivity of ~0.3 g h^−1^ due to the slow flow rates utilised^16–18^. This calls for the synthesis of leach-resistant SAC as well as flow reactors customized for SAC-catalysed reactions requiring high flow rates^19^.

**Fig. 1:**
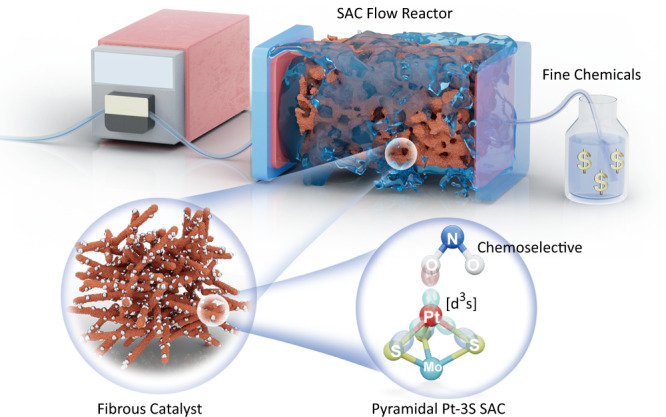
Schematic illustration. Continuous production of fine chemicals is demonstrated by a benchtop, SAC-modified flow reactor.

Herein, we report the fabrication of a SAC-supported MoS_2_/graphite felt flow stack that is built for fast-flow operation. Our SAC-catalysed flow stack can be used for the quantitative production of fine chemicals (~5.8 g h^−1^), including the synthesis of 28 examples of multifunctional anilines by the chemoselective reduction of nitroarenes on a Pt_1_-MoS_2_ catalyst. As shown in Fig. 1, the use of a 3D fibrous catalyst material allows the maximal exposure of single atom site to the reactants. Owing to the stability of the pyramidal Pt-3S structure, the fabricated Pt_1_-MoS_2_ catalyst is highly resistant toward metal leaching, thus allowing long-term operation of the flow reactor at a high flow rate (≥5 mL min^−1^) without performance degradation. At the reactor level, the influence of mass diffusion limitation and local turbulence inside the reactor was examined with the aid of computational fluid dynamics. The use of fast-flow reactor addresses the productivity bottleneck of SACs and paves the way for their applications in large-scale chemical production.

## Results

### Fabrication of the SAC fibrous catalyst module

Fast-flow reactors with turbulent flow paths require mechanically robust anti-leaching catalyst materials. We chose compressible graphite felt (GF) that is commonly used in highly electrochemically active redox flow batteries^20^ as the catalyst support. The GF was coated with hydrothermally grown MoS_2_ for the subsequent immobilisation of single-metal atoms, such as Pt, Fe, Co, Ni, and Cu (denoted as M_1_-MoS_2_-GF, where M_1_ stands for the active single atom)^21,22^.

**Fig. 2:**
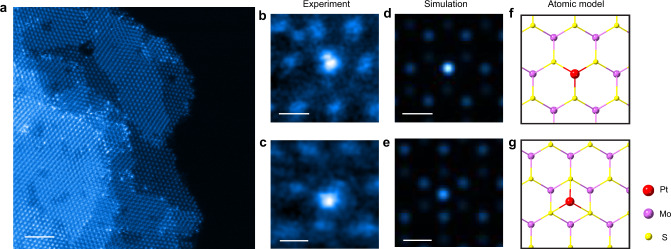
Identification of the Pt single atoms in the as-prepared catalyst. **a** Atomic-resolution STEM-HAADF image of Pt_1_-MoS_2_ (details in Supplementary Fig. 2); **b** Magnified view of a Pt atom adsorbed atop of Mo (dominant species, 93% in STEM images); (**c**) Pt atom in the hollow of the honeycomb structure (minor species, 7%). **d**, **e** The corresponding HAADF image simulations and **f**, **g** atomic models of the catalyst, **f** Pt directly adsorbed on top of Mo; **g** Pt adsorbed on hollow site of hexagon. Scale bar: a, 2 nm; b-e, 0.2 nm.

**Fig. 3:**
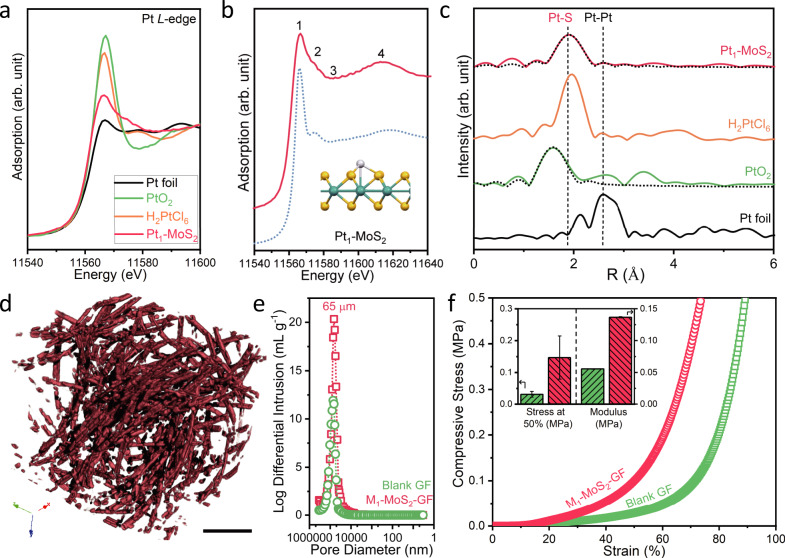
Characterisation of the M_1_-MoS_2_-GF catalysts. **a** Pt *L*_3_-edge XANES spectra; **b** Comparison between the experimental and calculated spectra using the pyramidal Pt-3S model (inset). The colour scheme used is as follows: light-cyan for Mo; yellow for S; white for Pt. Outer MoS_2_ atoms are omitted for the sake of clarity; **c** FT-EXAFS spectra of various catalysts. Dotted lines represent the fitting of the FT-EXAFS spectra; **d** False-coloured micro-CT image of the 3D structure of the M_1_-MoS_2_ array on graphite felt; **e** Mercury intrusion porosimetry and **f** Compressive strain–stress curves of blank GF and M_1_-MoS_2_-GF. Inset shows the comparison of the stress at 50% strain and compressive modulus at 15 ~ 20% strain. Error bars (SD) were presented from 5 individual tests. M = Co for (**d**–**f**); Scale bar: **d**, 100 µm.

Pt SAC shows good dispersion on the Pt_1_-MoS_2_-GF, the latter is considered as a catalyst module and can be packed into fuel cell type flow stacks and compressed to regulate porosity and flow dynamics. As shown in Fig. 2a and Supplementary Fig. 1 ~ 4, the atomic resolution scanning transmission electron microscopy (STEM) image confirms the uniform distribution of the individual Pt atoms on the MoS_2_ support, which are observed as bright spots overlapping the Mo column in the lattice structure of MoS_2_. A magnified view of the Pt atoms in Fig. 2b indicates that the majority of Pt atoms are situated on the Mo atop sites, while a small percentage (~7%) of atoms are located in the honeycomb structure (Fig. 2c and Supplementary Fig. 3)^23^. These observations are in good agreement with the simulated high-angle angular dark-field (HAADF) images and atomic models shown in Fig. 2d-g. Each Pt atom is covalently bonded to three S atoms to form a strongly bound pyramidal structure that resists leaching under fast flow conditions^24^. The coordination environment of Pt in the bulk structure was confirmed by X-ray absorption near-edge structure (XANES) and extended X-ray absorption fine structure (EXAFS) spectroscopy (Fig. 3). The Pt *L*_3_-edge XANES spectrum of Pt_1_-MoS_2_ exhibits a white line intensity much lower than that of PtO_2_ and the H_2_PtCl_6_ precursor (Fig. 3a), indicating a lower oxidation state of ~ +2 after annealing in H_2_^25^. The calculated and experimental XANES curves in Fig. 3b confirm the pyramidal Pt-3S coordination structure of our samples. A prominent Pt-S peak at ~1.8 Å was observed in the Fourier transform-EXAFS spectrum of the samples (Fig. 3c), which is also fitted with a coordination number of 3.7 in Supplementary Table 1. No metallic Pt-Pt peak at 2.6 Å is observed in the EXAFS spectra, indicating that all Pt_1_ atoms exist as isolated single atoms^26^. This result agrees well with the HAADF-STEM images where no nanoparticles are spotted. Supplementary Fig. 5 shows the STEM images and EXAFS spectra of Co_1_-MoS_2_-GF, further proving the robustness of our method.

We conducted X-ray microtomography (micro-CT) to examine the fibrous structure of the catalyst module^27,28^. As shown in Fig. 3d, the catalyst module is impregnated on carbon fibre with a convoluted, interconnected channel network, under compression, the pores are further constricted and local turbulent flow could be generated on the catalyst surface^20,27^. The outer surface of the fibre is considerably rough on the nanometre scale. Supplementary Fig. 6 and 7 show the scanning electron microscopy and micro-CT images of the MoS_2_ nanosheet array on the fibre, respectively. Very large pores are observed after the mercury intrusion porosimetry test, which reflects the surface roughness of the fibres (Fig. 3e and Supplementary Fig. 8). The pure GF and M_1_-MoS_2_ catalyst exhibit a typical pore size of approximately 65 µm with a porosity of 91–95%^29^. Such macropores allow fast diffusion kinetics under moderate pressure. This is distinct from the packed-bed reactors, in which the densely packed powdered catalyst requires higher pressure to obtain an equivalent flow rate^11^. Compressive strain-stress measurements were conducted to validate the mechanical robustness of the catalyst module. As shown in Fig. 3f, the M_1_-MoS_2_ modified catalyst exhibits a much higher compressive strength than pure GF (~200% increase in stress at 50% strain and ~150% increase in compressive modulus). The catalyst module can tolerate at least five independent compressive cycles with a 90% shape deformation without any structural degradation or powder detachment^30^. This proves the excellent adhesion of M_1_-MoS_2_ on the carbon support, thus minimising catalyst deactivation under the local stress concentration. Moreover, the surface of such a catalyst module is highly wettable, as confirmed by their water contact angle measurements (Supplementary Fig. 9). Further catalyst characterisations, including X-ray diffraction (XRD) and X-ray photoelectron spectroscopy (XPS), are shown in Supplementary Fig. 10 and 11.

### Flow cell performance for fine chemical production

A redox battery-type flow cell reactor was adapted for the SAC-catalyst module (Supplementary Fig. 12 ~ 14). Graphite felt, which is commonly used in vanadium redox flow batteries, was used as a support for the fabricated Pt_1_-MoS_2_ catalyst module, and its porous structure strengthened local turbulence and improved cell mixing and mass transport.

**Fig. 4:**
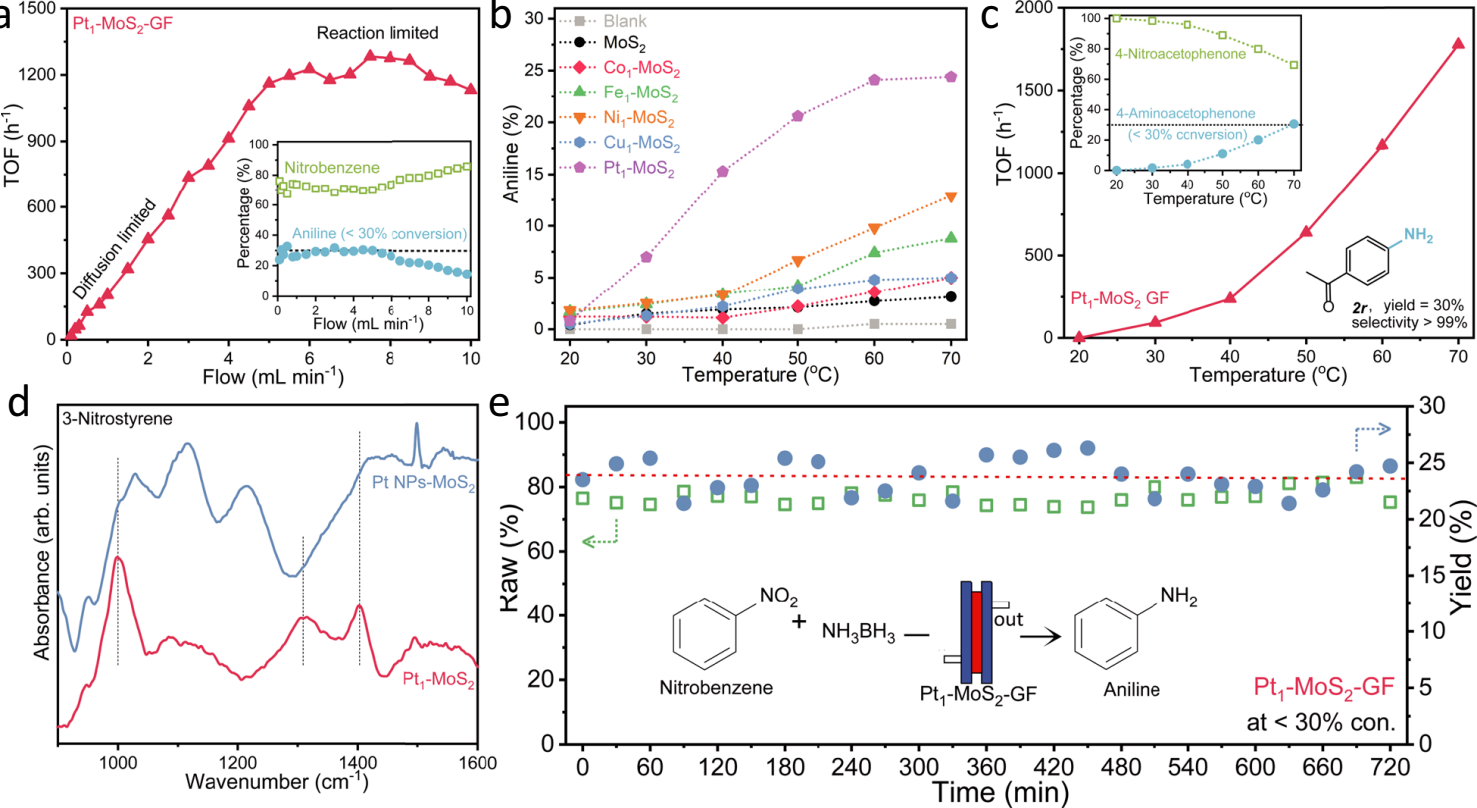
Selective nitroarene reduction in the flow setup. **a** Plot of TOF versus flow rate, showing the mass flow-limited regime and reaction-limited regime in the flow cell performance. Inset shows the percentage of reactant and product at various flow rates; **b** Catalyst screening of various SACs and controls in the reaction-limited, low-conversion regime; **c** Verification on the chemoselectivity of Pt_1_-MoS_2_ for the reduction of 4-nitroacetophenone. Inset shows the percentage of reactant and product at various temperatures, and the chemical structure of 4-aminoacetophenone (desired product); **d** In-situ DRIFTS spectra of Pt_1_-MoS_2_ and Pt nanoparticles on MoS_2_ after the hydrogenation of 3-nitrostyrene at 70 °C; **e** A 12 h on-stream demonstration of the Pt_1_-MoS_2_-GF catalyst in the low-conversion regime.

The synthetic utility of our flow reactor was demonstrated in the chemoselective reduction of nitroarenes to multifunctional anilines, which is industrially important in pharmaceutical and fine chemical production^31,32^. The chemoselectivity of SAC to retain reduction-prone functionalities has been previously demonstrated^33–38^. However, most of these tests were conducted under batch processes using high-pressure H_2_ gas, necessitating complex scale-up procedures. As shown in Fig. 4a, we examined the reduction of nitrobenzene using the Pt_1_-MoS_2_-GF catalyst module in a mixture of water and acetonitrile. The influence of mass diffusion limitation on the reactor performance was carefully studied by flow rate testing in the low conversion regime (<30%). It is seen that external mass diffusion limitation dominates the flow cell performance at low to medium flow rates, while the performance becomes reaction-limited at a flow rate of 7.5 mL min^−1^ or above. This gives a maximum turnover frequency (TOF) value of the active metal of approximately 1300 h^−1^. The conversion gradually decreases at higher flow rates owing to the decreasing residence time. Nevertheless, no side products are detected at the aforementioned flow rates, and the selectivity toward aniline remains above 99%. The degree of internal diffusion limitation (i.e., pore diffusion) is also verified by the Weisz-Prater criterion in Supplementary Table 3 and Supplementary Discussion, with negligible influence in the measured range.

Comprehensive catalyst screening on the SAC library was then conducted in the reaction-limited (i.e., 7.5 mL min^−1^) and low conversion regime (<30%) in Fig. 4b. Our Pt_1_-MoS_2_ outperforms other types of SACs and control samples. Detailed TOF analysis in Supplementary Fig. 15 shows comparable values to that of flow rate testing. Quantitative conversion to aniline over a wide temperature range (40–70 °C) could be achieved at a lower flow rate (1 mL min^−1^) using Pt_1_-MoS_2_ in the extra catalyst screening in Supplementary Fig. 16. The intrinsic chemoselectivity of Pt_1_-MoS_2_ was confirmed by the reduction of 4-nitroacetophenone to the desired 4-amino-acetophenone (99% selectivity) with an apparent activation energy barrier of 65 kJ mol^−1^ in Fig. 4c. This indicates that the thermodynamic selectivity of the reaction depends on the preferred interaction between the nitro group and atomic metal site, rather than on kinetically controlled reactivity^33^. The stability tests were conducted at low conversions (<10% and <30%) for a continuous 12 h operation at 70 °C. No significant decay was observed in Fig. 4e and Supplementary Fig. 17, despite minor fluctuations in yield due to temperature fluctuation and GC-MS sampling. Further stability examination was conducted in the quantitative conversion regime for 24 h to demonstrate the steady production of valuable products (Supplementary Fig. 18). We have also performed a comprehensive characterisation of the spent catalyst by XRD, XAS and STEM in Supplementary Fig. 19 and 20 to prove the single atom nature after reaction. Importantly, the productivity of our reactor can be significantly enhanced by operating at a higher reactant concentration (0.2 M). We can achieve a maximum aniline productivity of 5.8 g h^−1^, corresponding to a TOF value of approximately 8000 h^−1^ in Supplementary Fig. 21, which is much higher than the reported values of 0.02–0.07 g h^−1^ for the same SAC-mediated reaction using batch reactors^33–38^.

**Fig. 5:**
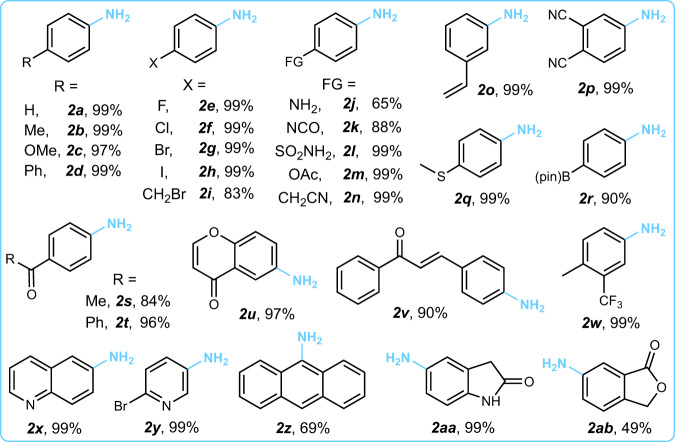
Reaction scope. Substrate scope of the Pt_1_-MoS_2_-GF catalysed nitroarene reduction in the quantitative conversion regime. The yields obtained through gas chromatography-mass spectrometry at the optimal conditions are shown here. Further details are provided in Supplementary Fig. 22.

The scope of the Pt_1_-MoS_2_ catalysed nitro-reduction can be extended to other sensitive functionalities. The flow reactor is operated at low flow rate (1 mL min^−1^) for quantitative conversion of the pricey building blocks. As shown in Fig. 5, Supplementary Fig. 22 and 23, multifunctional amines with alkyl (**2a**, **2b**)-, aryl- (**2d**), alkoxyl- (**2c**), halogen- (**2e**–**2i**, **2w**), amino- (**2j**), sulfonamide- (**2** **l**), ester- (**2** **m**), methylthio- (**2q**), and boronic acid pinacol ester (**2r**) substitutions at the para position can be efficiently synthesised (65%–99% yield). The most commonly occurring and versatile functionalities, including those with potentially reducible functional groups, such as ketones (**2s**-**2v**), alkenes (**2o**, **2** **u**, **2** **v**), nitriles (**2n**, **2p**), isocyanate (**2k**), and quinoline (**2x**), are well tolerated in our protocol (84%–99% yield), highlighting its remarkable chemoselectivity. Notably, 3-aminostyrene (**2o**, 99% yield), an important feedstock chemical^33^, can be efficiently produced by the reduction of 3-nitrostyrene. Moreover, 6-nitrochromone (**2** **u**) and 4-nitrochalcone (**2** **v**) bearing multiple reducible groups (ketone and internal alkene) can be selectively reduced to their corresponding amines, which are usually difficult to synthesise using previous methods owing to side reactions^39^. It is worth pointing out that conventional gas-phase hydrogen reductions with noble metal catalysts suffer from low selectivity at high temperatures. We also compared the chemoselectivities of the Pt_1_-MoS_2_ catalyst and commercially available 10% Pt/C catalyst using our flow setup (Supplementary Fig. 24**)**. Here, results show that Pt/C catalyst has very poor selectivity with only 10% yield. Continuous-flow production can also be applied to anthracene (**2z**) and many heterocycles, including pyridine (**2** **y**), quinoline (**2x**), oxindole (**2aa**), and phthalide (**2ab**). Finally, our continuous-flow protocol can be extended to chemoselective oxidation of sulfides with similar productivity enhancement using a Co_1_-MoS_2_ catalyst module (Supplementary Fig. 25 ~ 29), suggesting its universal applicability to address the conversion bottleneck in SAC-catalysed reactions.

### Mechanistic study of chemoselectivity

The chemoselectivity of Pt_1_-MoS_2_ catalyst for nitro-reduction reaction is proposed to originate from the preferential adsorption of nitro groups on Pt single atom. The mechanistic study was conducted using in-situ diffuse reflectance infrared Fourier transform spectroscopy (DRIFTS) of 3-nitrostyrene under a gaseous mixture of H_2_ and N_2_ at 70 °C on Pt_1_-MoS_2_ and a control sample of Pt nanoparticles on MoS_2_^38,40^. The adsorption mode of alkene depends on the substrate, it shows a weak adsorption via *π*-bonded mode on isolated single atoms in comparison to strong ethylidyne (tridentate) or di-*σ* (bidentate) configuration on nanoparticles. By contrast, the adsorption of nitro functionality prefers a monodentate “end-on” configuration^41^. Three absorption bands were observed at 1000, 1310, and 1402 cm^−1^ in the Pt_1_-MoS_2_ spectra (Fig. 4d, Supplementary Fig. 30 and 31). These bands were assigned to the nitro group adsorbed via the “end-on” configuration and its reduced intermediates (phenylhydroxylamine & nitrosobenzene)^41^. This suggests that the reaction proceeds through a direct hydrogenation route to form aniline, rather than the condensation route to form azobenzene^42^. For the control sample, we observed two additional bands at 1116 and 1214 cm^−1^, which are probably related to the planar binding configuration of the alkene groups. We have also conducted the CO-DRIFT and CO stripping experiments in Supplementary Fig. 32, where CO adsorption peak was not detected on Pt_1_-MoS_2_^33^. In contrast, a strong CO stripping peak was observed for the 10% Pt/C control sample.

**Fig. 6:**
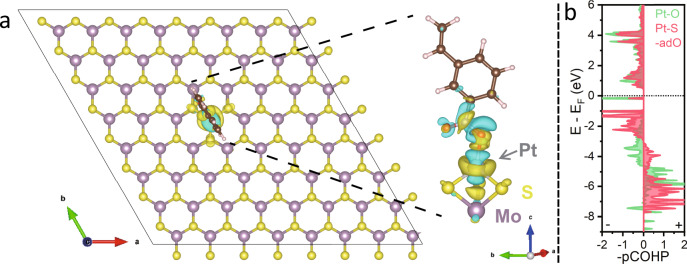
DFT calculations. **a** Differential charge density of the optimized “end-on” configuration of 3-nitrostyrene on Pt SAC at the Mo atop site, showing electron accumulation (yellow) and depletion (cyan) regions. The color scheme used: purple for Mo, yellow for S, white-grey for Pt, red for O, light-blue for N, brown for C and white-pink for H; **b** The -pCOHP curves for 3-nitrostyrene adsorption on Pt SAC at the Mo atop site (green: Pt-O bond; red: nearby Pt-S bond, denoted as Pt-S-adO).

The preferential binding of nitro functionality on Pt is supported by density functional theory (DFT) calculations on the Pt active site^43–45^. There are two possible binding sites for Pt, either atop of the Mo or on the hollow of the honeycomb. STEM reveals that the atop configuration is the dominant binding site (Fig. 2b), and this is also supported by the DFT calculation of formation energies (Supplementary Fig. 33). Unlike metallic Pt (111) surface, the Pt SAC exhibits a lower d-band center in Supplementary Fig. 34 and Supplementary Table 4. This is supported by the positive charge (Pt^*δ*+^) of Pt_1_ on MoS_2_ in Bader charge analysis compared to Pt^0^ in the Pt (111) facet. Differential charge analysis in Fig. 6a and Supplementary Fig. 35 reveals a highly directional charge distribution along the *z-*axis that may benefit the adsorption of polar functionality^41^. This is supported by the projected crystal orbital Hamilton populations (pCOHP) of Pt and adjacent S atoms in Fig. 6b, Supplementary Fig. 36 and 37^31,46^. The Pt^2+^ metal center (*d*^8^*s*^0^) in Pt_1_-MoS_2_ forms [*d3s*] hybrid orbitals involving the 6 *s*, 5*d*_x2-y2_, 5*d*_xy_ and 5*d*_yz_ orbitals, and these hybridize with the 3*p* orbitals of S atoms, leaving a half-filled orbital along the *z-*axis; the latter bonds with electron-deficient nitro functionality through the Pt hybrid 5*d*6*s* – O 2*p*_z_ bonding (Supplementary Table 5). In contrast, the 6*s* electrons of Pt^0^ (*d*^9^*s*^1^) in bulk Pt (111) are paired to form the Pt-Pt bond, this makes the bonding with nitro functionality adopting an “end-on” configuration weaker. The integration of -pCOHP below the Fermi level is a quantitative measure of bonding strength^47^. For the Pt-O bonding on Pt SAC, the integration value is roughly 10 fold greater than that on Pt (111) facet. This agrees well with adsorption energies of −1.23 versus −0.66 eV for “end-on” adsorption on Pt_1_-MoS_2_ and Pt (111) facet in Supplementary Fig. 38 and 39.

### Understanding flow cell performance

The flow cell performance depends not only on the intrinsic activity of catalyst, but also on the mass transfer and heat exchange efficiency inside the reactor^48^. Particularly, the operation in quantitative conversion regime usually suffers from severe mass diffusion limitation (Fig. 4a), and improvement in reactor performance is usually achieved by optimizing the fluidic behavior to enhance mass transfer kinetics.

We conducted computational fluid dynamics (CFD) calculations of a reconstructed 3D model derived from the micro-CT images (Fig. 3d) of the SAC catalyst module to study the connection between microstructure and flow behaviours^20^. As shown in Supplementary Fig. 40a, the highly irregular carbon fibres in the catalyst module induces local turbulence and sharp velocity gradient near the solid catalyst surface, creating catalytically active hot spots for chemical conversion. Previous studies of redox flow batteries reveal that electrochemical conversion occurs on the hot spots in compressed carbon fibres^20^. The presence of fluidic hot spots is confirmed by greater values in turbulence kinetic energy (TKE) than the open area in Supplementary Fig. 40b, an indication of the strength of turbulence by measuring the root-mean-square velocity fluctuation^46,49^. Preferred flow paths are identified through the porous structures, where the flow velocity gradient increases sharply in the narrow void space between carbon fibres^18^. A greater number of hot spots are created at a higher flow rate, e.g., 50 mL min^−1^ versus 0.1 mL min^−1^ (Supplementary Fig. 41**)**^20^. Reynolds number (*Re*) is a dimensionless mass transfer coefficient that is used to characterize the flow behavior^10^. As expected, the strong correlation between Reynolds number and the TOF value of Pt_1_-MoS_2_ is observed in the diffusion-limited regime in Supplementary Table 2, however, the TOF value becomes insensitive to the Reynolds number when it is reaction controlled. Additional CFD results on the velocity and TKE contours at various planes and directions further indicate the complexity of the local flow field inside real catalyst compartment (Supplementary Fig. 42 ~ 48).

The thermal management and how compression improves performance for the reactor are discussed and shown in Supplementary Fig. 49 ~ 52. Going forward, more work is needed to customize reactor design for SACs. Beyond tuning the fluidic behavior inside the reactor, local variations in porosity and tortuosity, as well as the catalytically active surface area (i.e., wettability and liquid-solid interface) are among the important factors to determine the reactor performance^20^. However, such understanding has not been established in the area of SACs.

In conclusion, we have successfully demonstrated the SAC-catalysed chemoselective reduction of nitro compounds to produce multifunctional anilines and other fine chemicals using a bench-top flow cell. A high TOF (>8000 h^−1^) and productivity (5.8 g h^−1^ of aniline) were recorded for this reaction using a Pt_1_-MoS_2_ catalyst module. In-situ DRIFTS and DFT calculations confirm that the chemoselectivity originates from the pyramidal Pt-3S structure of the catalyst, which prefers the “end-on” adsorption of the nitro groups in organic molecules. The pyramidal Pt-3S coordination structure binds strongly to MoS_2_ and prevents leaching during the flow reaction, resulting in highly stable performance in the continuous operation at low and quantitative conversions. The successful demonstration of exceptionally high quantitative conversion in SAC-catalysed reactions operated under fast flow condition paves the way for their application in liquid phase synthesis of fine chemicals.

## Methods

### Synthesis of Pt_1_-MoS_2_-GF catalyst

MoS_2_ nanosheets were grown on GF by a conventional hydrothermal method^21^. To prepare the Pt_1_-MoS_2_ catalyst, the as-prepared MoS_2_-GF was immersed in 100 mL of H_2_PtCl_6_·6H_2_O aqueous solution (1 mM) at 80 °C for 2 h. Subsequently, MoS_2_-GF was rinsed with DI water and ethanol and dried at 60 °C. The modified material was then annealed at 300 °C for 2 h under a 95%/5% Ar/H_2_ mixture to obtain Pt_1_-MoS_2_-GF with a Pt loading of ~0.2 wt%^22^.

### Chemoselective reduction of nitroarenes in the flow setup

The flow reactor was assembled using one piece of Pt_1_-MoS_2_-GF (4 × 4 cm^2^). A pre-mixed stock solution of 0.10 M nitroarene and 0.05 M ammonia borane (0.5 equiv.) in an acetonitrile/H_2_O mixture (5:1, v/v) was supplied to the flow reactor by a peristaltic pump at the desired flow rate (7.5 mL min^−1^) and heated to the desired temperature (20 to 70 °C). The clear solution was collected after a stable period of 30 min for each temperature or flow rate. The conversion and yield were monitored by gas chromatography-mass spectrometry (GC-MS). Details of the experimental setups (including the flow cell setup, stability test, other types of flow reactions, in-situ DRIFTS, material characterisations, DFT and CFD calculations) can be found in the Supplementary Materials.

## Supplementary information

Supplementary Information

## Data Availability

All data are available from the authors upon reasonable request.
