# Supplementary material for: Addressing the quantitative conversion bottleneck in single-atom catalysis
Source: Nat Commun. 2022 May 19;13:2807. doi: 10.1038/s41467-022-30551-w (PMC9120447; doi:10.1038/s41467-022-30551-w)
Supplement: Supplementary file 1 — Supplementary Information [file 41467_2022_30551_MOESM1_ESM.pdf]

Addressing the Quantitative Conversion Bottleneck in Single-Atom Catalysis

Zhongxin Chen,+ Jingting Song,+ Rongrong Zhang,+ Runlai Li, Qikun Hu, Pingping Wei, Shibo Xi, Xin Zhou, Phuc T. T. Nguyen, Hai M. Duong, Poh Seng Lee, Xiaoxu Zhao, Ming Joo Koh, Ning Yan and Kian Ping Loh*

+ These authors contributed equally to this work. Correspondence and requests for materials should be addressed to K. P. Loh (chmlohkp@nus.edu.sg)

Supplementary Methods

Flow cell setup. The flow cell was customized with two stainless-steel cover plates, two copper plates, two monopolar graphite plates with a parallel path design for organic solvent distribution (depth of flow fields: 0.80 mm; length per flow field: 40 mm; number of flow field lines: 15; total length of flow fields: 600 mm) on one side and a closed configuration on the other side, a 4×4 cm^2 graphite felt catalyst, PTFE gaskets, O-rings and plug-in adaptors for silicone tubings (3.2 mm O.D., 1.6 mm I.D.). Flow rate was controlled by a Watson Marlow 120S peristaltic pump and the actual flow rate was determined by measuring cylinder. Temperature was controlled by two heating rods with thermocouple and digital controller. The heating rods were plugged into the stainless-steel plates to avoid direct contact with organic solvent and catalyst. Temperature calibration was done with external thermocouple.

Synthesis of Pt_1-MoS_2-GF catalyst. MoS_2 nanosheets were directly grown on O_2-treated, hydrophilic soft graphite battery felt (AvCarb G200, **GF**, 6.5 mm thickness, areal weight 480 g m^{-2}) by a simple hydrothermal method.[1] In a typical synthesis of MoS_2 on GF (denoted as MoS_2-GF), $Na_2MoO_4 \cdot 2H_2O$ (18 mmol, 4.355 g) and thiourea (48 mmol, 3.648 g) was dissolved in 100 mL of deionized water. After gentle stirring for 30 min, the solution was then transferred to a 250 mL Teflon-lined stainless-steel autoclave with a piece of graphite felt (4×4 cm^2). The autoclave was sealed and heated at 190 °C for 24 h in an oven and then cooled down to room temperature naturally. Finally, the product was taken out, rinsed with deionized water and ethanol several times and dried at 60 °C in air. The loading of MoS_2 nanosheet on GF was ~ 45 mg cm^{-2} by weight difference and ICP-OES. To prepare Pt_1-MoS_2 catalyst, as-grown MoS_2 materials were immersed into 100 mL of $H_2PtCl_6 \cdot 6H_2O$ aqueous solution (1 mM) at 80 °C for 2 h, and then rinsed with DI water and ethanol before dried at 60 °C. The modified material was loaded into a quartz tube mounted inside a tube furnace under 95%/5% Ar/H_2 mixture and heated at 300 °C for 2 h at 10 °C min^{-1}.[2] The Pt loading on MoS_2 was determined as ~ 0.43 wt% (~ 0.21 wt% including the weight of GF) from ICP-OES. Co_1, Fe_1, Ni_1 or Cu_1-MoS_2-GF were prepared using $CoCl_2 \cdot 6H_2O$, $FeCl_3 \cdot 6H_2O$, $NiCl_2 \cdot 6H_2O$ or $CuCl_2 \cdot 6H_2O$ as precursor at a higher concentration of 10 mM. The Co, Fe, Ni and Cu loading on MoS_2 catalysts were determined as ~ 0.22, 0.27, 0.28, 3.13 wt% on GF, respectively. Control samples of 10 wt% Pt/C on GF was prepared by spray-coating method at the same Pt loading.

Chemoselective reduction of nitrobenzene in flow setup. Typically, 1 piece of Pt_1-MoS_2-GF (4×4 cm^2) was loaded into the flow cell. A pre-mixed stock solution of 0.10 M nitrobenzene and 0.05 M ammonia borane (0.5 equiv.) in acetonitrile/H_2O mixture (5:1, v/v) was supplied to the flow cell by a peristaltic pump and the flow rate was regulated to the desired value (75 ≈ 7.5 mL min^{-1}) for measurement in the reaction-limited regime. The flow cell was heated to desired temperatures (r.t. to 70 °C) with the heating unit. Clear solution was collected after a

stable period of 30 mins for each temperature. Conversion and yield were monitored by GC-MS. Other nitroarenes or catalysts were examined by the same method. 5% of DMF is added for those substrates with poor solubility in acetonitrile/H_2O mixture. For measurement in quantitative conversion, the usage of nitrobenzene was reduced to 0.025 M at a flow rate of 1 mL min^{-1}.

Stability test of nitrobenzene reduction. The stability test at 30% conversion was conducted at 70 °C using 0.10 M nitrobenzene and 0.05 M ammonia borane (0.5 equiv.) in acetonitrile/H_2O mixture (5:1, v/v) at a constant flow rate of 1 mL min^{-1}. Clear solution was collected every 30 mins for a total measurement period of 12 h. The usage of nitrobenzene was increased to 0.20 M for stability test at 10% conversion for 12 h. Stability test at quantitative conversion was also conducted for 24 h using 0.025 M nitrobenzene at a flow rate of 1 mL min^{-1}. Rate capability test was conducted at the 20th hour under dynamic flow rates of 0.1 ~ 20 mL min^{-1}. The flow rate was resumed to normal (1 mL min^{-1}) after rate capability testing and the next sample was collected after a stable period of 30 mins. Conversion and yield were monitored by GC-MS.

***In-situ* DRIFTS characterisation.** DRIFTS measurements were performed on a Thermo Scientific Nicolet iS50 instrument with an Hg-Cd-Te (MCT) detector and a Praying Mantis high temperature reaction chamber with ZnSe windows. Powder samples were used due to poor IR signal on carbon support. Initial heating was conducted under N_2 at 20 mL min^{-1} from room temperature to 70 °C at 10 °C min^{-1}. Background spectrum was acquired until stable. Nitrobenzene or 3-nitrostyrene was then introduced to the reaction cell by a mixture of N_2 and H_2 (2:1 v/v, 30 mL min^{-1}). DRIFT spectra were collected for a continuous period of 30 min. The reaction cell was then purged by pure N_2 at 20 mL min^{-1} for another 30 min and then cooled down to room temperature.

Material characterisation. The following equipment were used: STEM (JEOL ARM200F equipped with ASCOR probe corrector, Oxford X-Max 100TLE, at 80 kV), SEM/EDS (JEOL JSM-6701F), XPS (Quantera II, monochromatic Al K_a), XRD (Bruker D2 & D8), GC-MS (Agilent 5975 C inert MSD with triple-axis detector), UV-Vis (Shimadzu UV-3600), ICP-OES (Perkin Elmer Avio 500), thermal imaging (FLIR One), mechanical test (Instron 5500), mercury intrusion porosimetry (AutoPore V 9600), water contact angle (AST Products VCA Optima), X-ray tomography (Skyscan 2211). X-ray absorption measurements were carried out at Singapore Synchrotron Light Source (SSLS), X-ray Absorption Fine structure for catalysis (XAFCA) beamline.[3] Data analysis and simulation were carried out on Athena, Artemis, and Hephaestus (Version 0.9.23).[4]

Computational Fluid Dynamics. The catalyst structures (ASCII-*.stp file converted from the raw files from Skyscan 2211) were imported into the CFD software ANSYS to analyze the porous nature and to simulate the flow behavior. The CFD simulations were conducted with the finite volume method (FVM) according to Schröder *et al.* (*ACS Appl. Energy Mater.* **2020**, *3*, 4384).[5] To obtain a well-defined surface description for calculation, a cuboid with the dimensions 0.2 × 0.2 × 0.1 mm^3 was subtracted from the original structure (0.7 × 0.7 × 0.7 mm^3) from X-ray tomography, and subjected to modifications to eliminate pierced faces, close proximity faces, and nonmanifold edges and vertices. The size of the actual model (0.25 × 0.25 × 0.13 mm^3) used was slightly larger than the subtracted model. For the flow simulations, a constant density of the fluid of 786 kg m^{-3} and a constant dynamic viscosity of 0.369 mPa·s from acetonitrile were assumed. The inlet boundary condition was set to a constant fluid velocity of 2 mL min^{-1} (equivalent to the flow rate of 2 mL min^{-1}), ambient pressure was set at the outlet boundary, and the no-slip condition was set at the catalyst surface. Flow simulations were performed in the X and Y directions, as well as at a higher (50 mL min^{-1}) or a lower flow rate (0.1 mL min^{-1}). For the sake of comparison, flow simulation was also performed in a blank model with the same dimensions.

DFT Computations. The first-principles calculations were performed with density functional theory (DFT) as implemented in Vienna *ab-initio* Simulation Package (VASP).[6,7] The generalized gradient approximation (GGA) in the Perdew-Burke-Ernzerhof (PBE) format,[8] the projector-augmented wave (PAW) method[9] and a plane-wave basis with the kinetic cut-off energy of 500 eV are employed in all calculations. G-centered 1 × 1 × 1 k mesh and 3 × 3 × 2 k mesh were used in geometry optimization process and static calculations respectively. The Fermi scheme was employed for electron occupancy with an energy smearing of 0.1 eV. The energy (converged to 1.0×10^{-5} eV $atom^{-1}$) and force (converged to 0.02 eV $Å^{-1}$) were set as the convergence criterion for geometry optimization. The projected Crystal Orbital Hamilton Population (pCOHP) method was used to visualize and analyze chemical bonding between catalysts and adsorbates with MP-centered k-point of 5 × 5 × 5, and the computer program Local Orbital Basis Suite Toward Electronic-Structure Reconstruc-tion (LOBSTER) was used to process the pCOHP calculation results.[10,11]

Modelling of Pt_1-MoS_2 slab. The MoS_2 (001) surface was obtained by cutting the MoS_2 bulk along the {001} direction. A Pt-modified single layer MoS_2 nanosheet ($Pt_1Mo_{64}S_{128}$) was employed as the catalyst model. The Pt atom was located on top of the Mo column on the surface of MoS_2 (Pyramidal Pt-3S SAC).A vacuum layer as large as 20 Å was used along the *c* direction normal to the surface to avoid periodic interactions. In all structural optimization calculations, all of the atoms were allowed to relax. Other SAC-modified MoS_2 models were obtained by a similar method. For pCOHP calculations, a smaller mesh is employed.

Supplementary Discussion

The influence of external diffusion limitation was examined by flow rate testing in **Fig. 4a**. We use the Weisz-Prater criterion to examine the influence of internal diffusion limitation:

$$N_{W-P} = \frac{\Re R_p^2}{C_s D_{eff}} \leq 3\beta$$

$$\text{and } \eta = \frac{3}{R_p^3}\int_0^{R_p}[1 - \beta(1 - r/R_p)^n]r^2 dr$$

where $\Re$ is the reaction rate per volume of catalyst, R_p is the catalyst particle radius (~ 5 μm for the fibre from SEM), C_s is the reactant concentration at the catalyst surface (0.1 M of nitrobenzene), and D_{eff} is the effective diffusivity, η is the effectiveness factor, n is the reaction order and β is the quantity defined above.

The D_{eff} can be calculated by

$$D_{eff} = \frac{D\varepsilon_t\delta}{\tau}$$

where D is the diffusion coefficient of liquid filling the pores (D = 0.684 × 10^{-9} m^2 s^{-1} for nitrobenzene at 293 K in bulk liquid), ε_t is the porosity available for the transport (95% for the open channel in graphite felt), δ is the constrictivity ($\delta = \frac{\sqrt{\max cross{-}section \times \min cross{-}section}}{mean\ cross{-}section}$ ≈ 1 for such liquid diffusion in open channel, *i.e.*, negligible hindrance for diffusion), and τ is the tortuosity of our graphite felt ($\tau = \frac{L_{average\ length\ of\ fluid\ path}}{L_{geometrical\ length}}$, it has a typical value of 1.05 ~ 1.09 for 23 ~ 58% compression, reference value from *Energies* **2019**, *12*, 313). Hence, D_{eff} is calculated as ~ 0.619 × 10^{-9} m^2 s^{-1}. Knudsen flow is not applicable to our system as the open channel (65 μm) is significantly larger than the typical Knudsen regime (< 100 nm).

The $\Re$ value is calculated as $\Re = \frac{C_s \times v \times conversion}{V_{cat}}$, where υ is volumetric flow rate (7.5 mL min^{-1}), V_{cat} is the catalyst volume ($V_{cat} = V_{reactor} \times (1 - \varepsilon_t) = 0.05\ cm^3$). At a typical conversion of 25% at 70 °C at 7.5 mL min^{-1}, the $\Re$ is 0.0625 M s^{-1}.

Therefore, the left part of Weisz-Prater criterion is,

$$N_{W-P} = \frac{\Re R_p^2}{C_s D_{eff}} = \frac{0.0625\ M\ s^{-1} \times (5 \times 10^{-4}\ cm)^2}{0.1\ M \times 6.19 \times 10^{-6}\ cm^2 s^{-1}} = 2.52 \times 10^{-2}$$

For the right part of Weisz-Prater criterion (β value), a given effectiveness factor (η) is assumed. The correlation between η and β in our system (R_p = 0.5 μm, n = 0, 1 or 2) is provided in **Supplementary Table 3**.

From the above calculations, we can conclude that pore diffusion limitation is **negligible** (*i.e.*, the Weisz-Prater criterion is satisfied) for our system. This also agrees well with our previous discussion, where the mass diffusion limitation is mainly attributed to external mass diffusion from bulk solution (*i.e.*, can be addressed at high flow rates).

Supplementary Figures

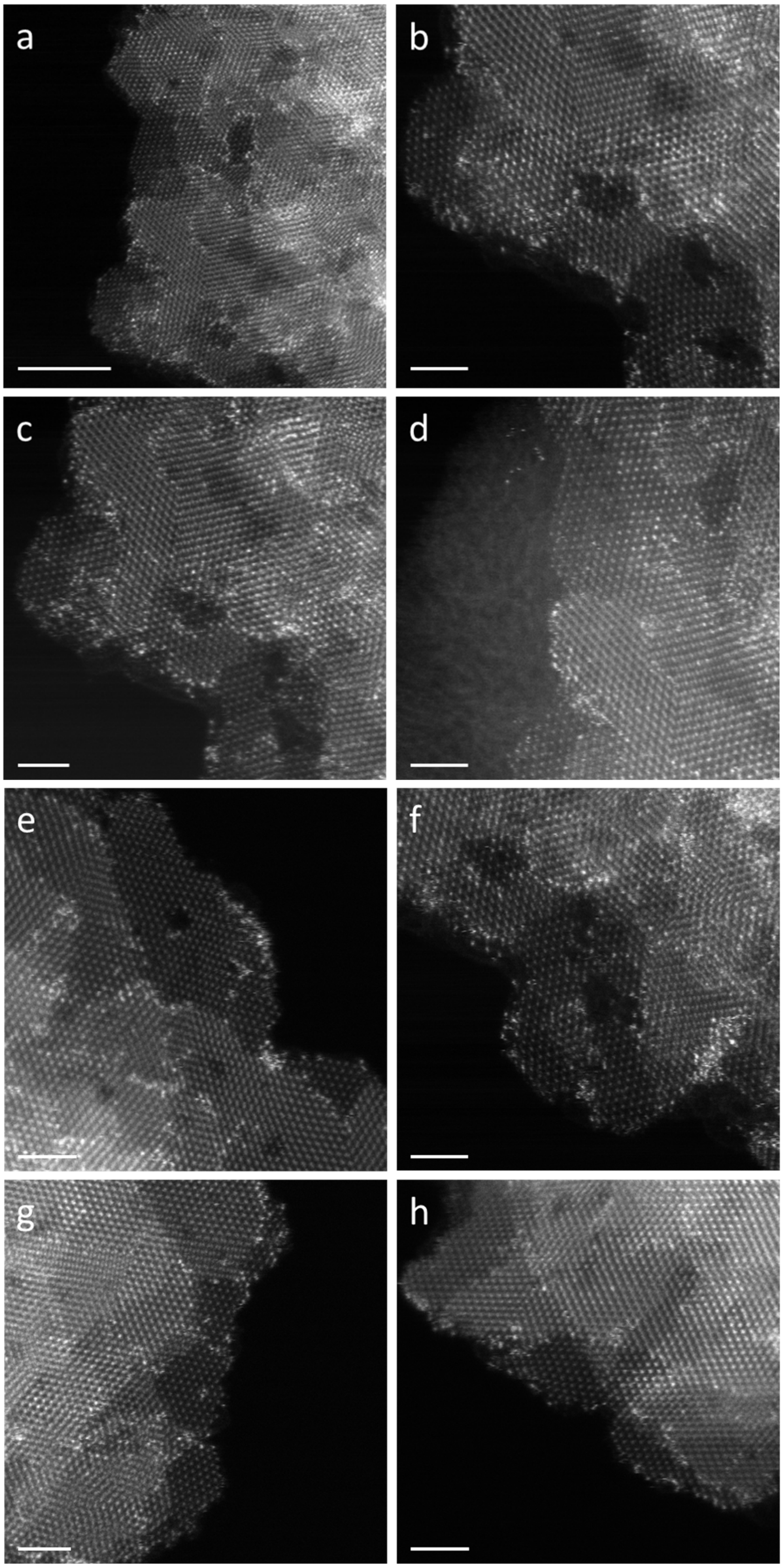

Supplementary Figure 1. (a-h) Representative STEM images of Pt_1-MoS_2-GF. Scale bars: a, 5 nm; b-h, 2 nm.

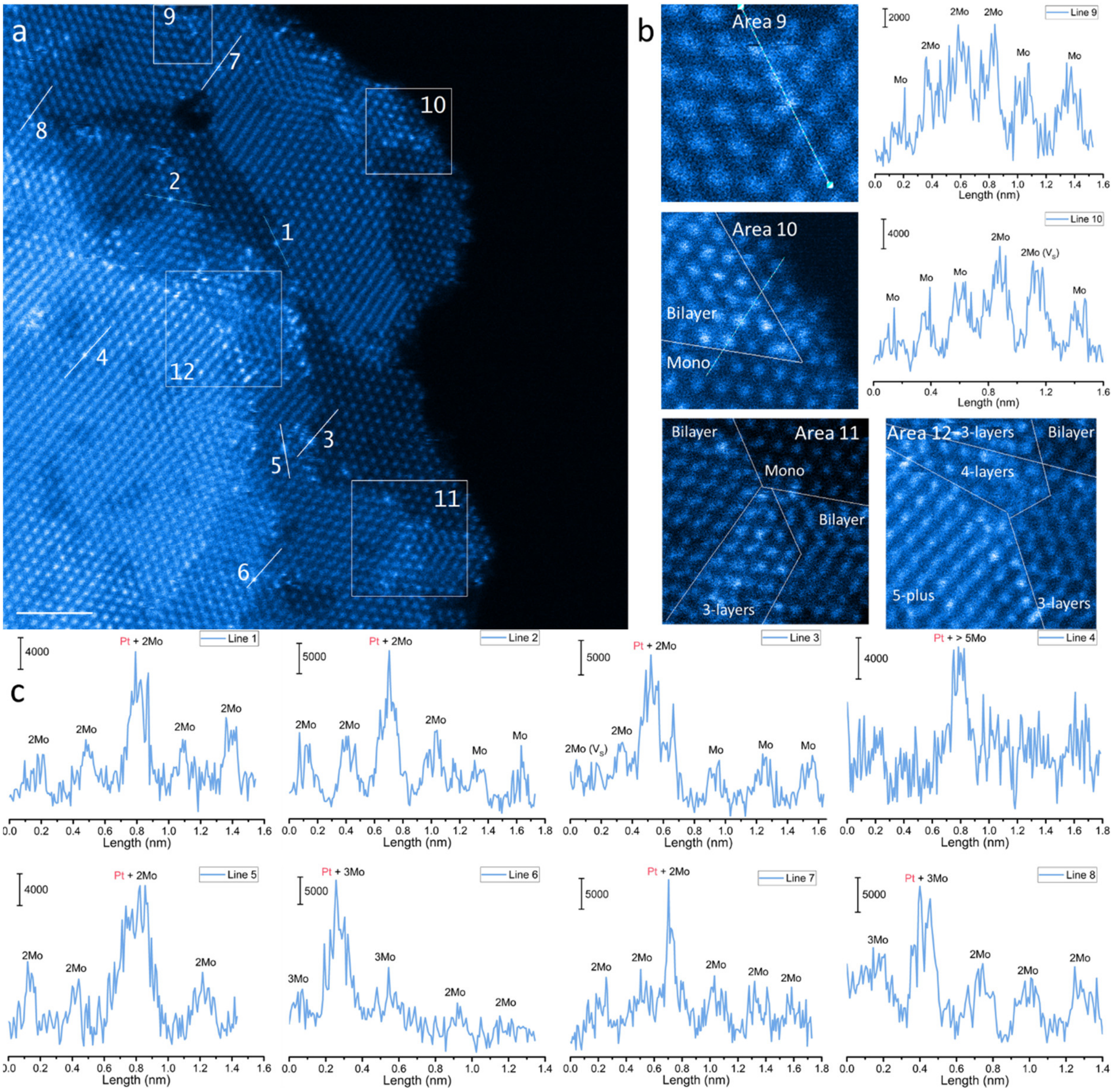

Supplementary Figure 2. (a) STEM evidence of the Pt single atoms, reproduced from **Fig. 2a** with an area of > 250 nm^2. (c) Line profiles show these are Pt single atoms rather than Pt dimers or trimers (locations 1-8), and (b) the domains in area 9-12 are actually different stacking of two or more layers of MoS_2. Images are false-colored for better contrast. Scale bar: 2 nm. V_S indicates the possible sulfur defects in the bilayer structure.

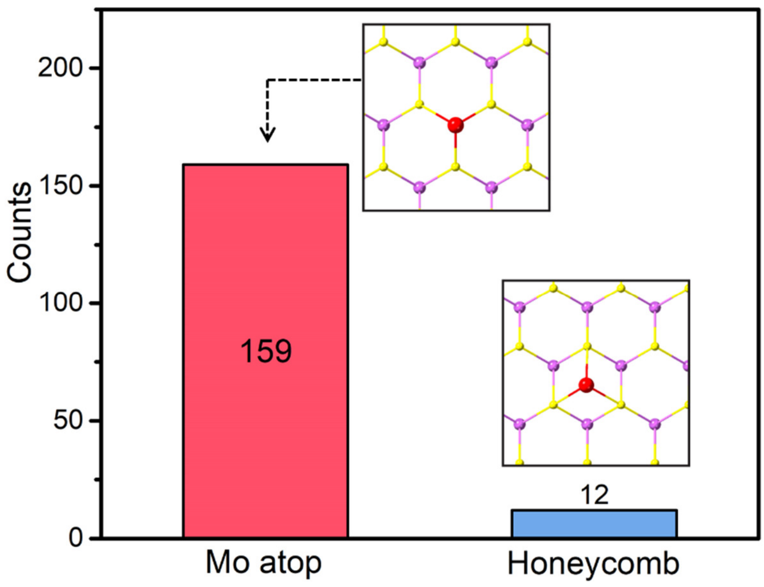

Supplementary Figure 3. Statistics of the single atom configurations in Pt_1-MoS_2-GF from STEM. Inset shows the corresponding atomic structures.

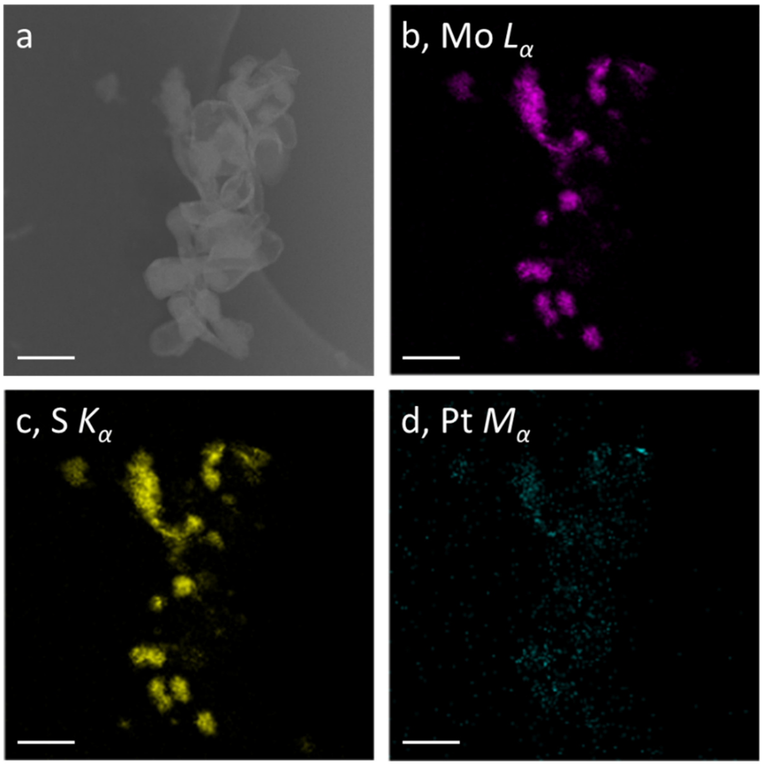

Supplementary Figure 4. (a-d) STEM EDS mapping of Pt_1-MoS_2-GF. Scale bars: a-d. 200 nm.

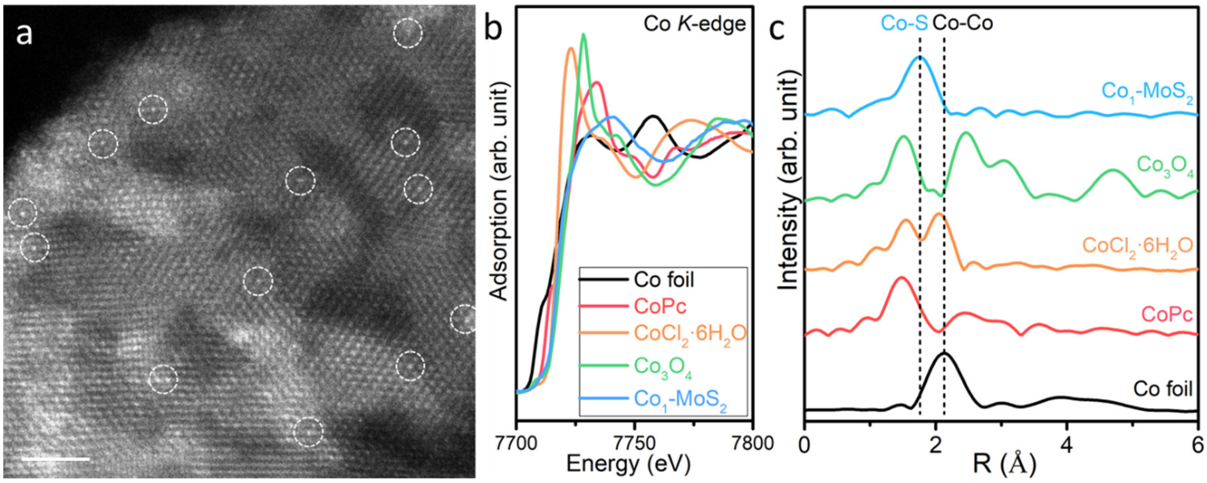

Supplementary Figure 5. (a) Atomic-resolution STEM-HAADF image of Co_1-MoS_2-GF; (b) Co *K*-edge XANES spectra; and (c) FT-EXAFS spectra of various catalysts.

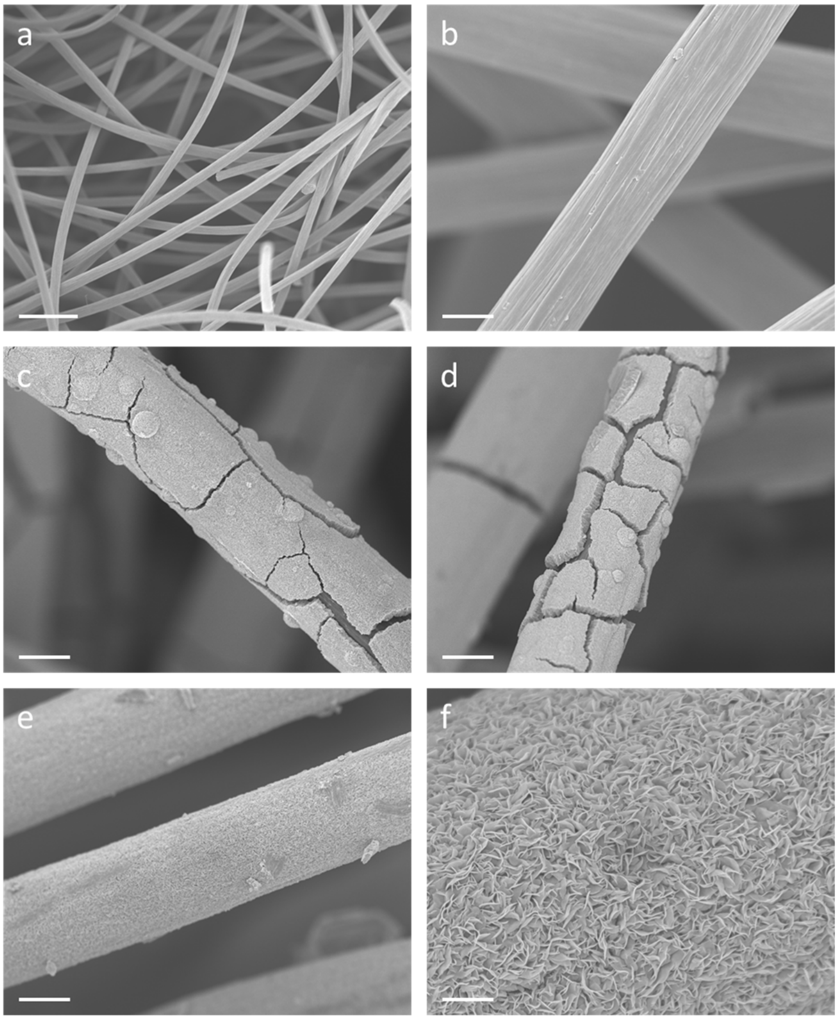

Supplementary Figure 6. SEM images of (a,b) blank graphite felt, (c) MoS_2-GF, (d) Co_1-MoS_2-GF and (e, f) Pt_1-MoS_2-GF at different magnifications. Scale bar: a, 50 μm; b-e, 5 μm; f, 1 μm.

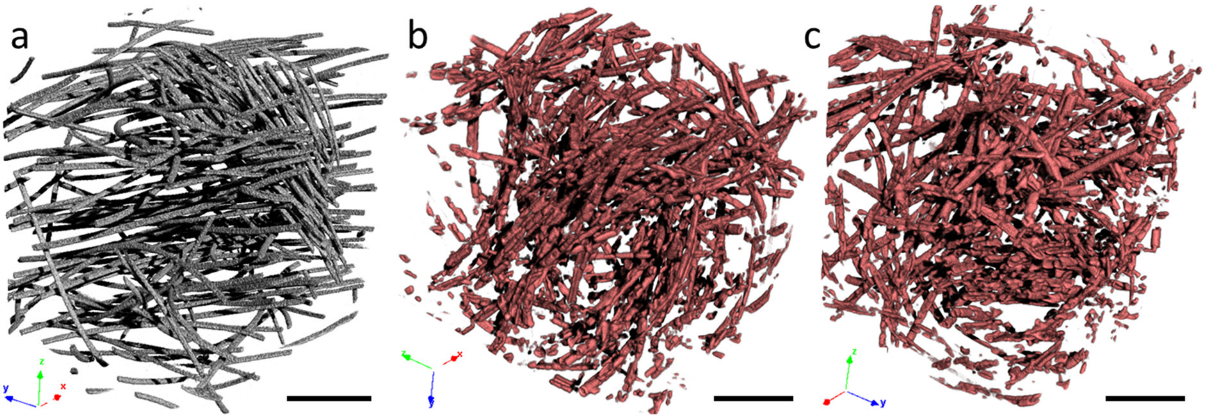

Supplementary Figure 7. False colored X-ray tomography images of (a) blank GF and (b, c) M_1-MoS_2-GF at various angles (M = Co). Scanning dimension: 0.7 × 0.7 × 0.7 mm^3. Point resolution: 200 nm. Samples were prepared by wire cutting. Scale bar: 100 μm.

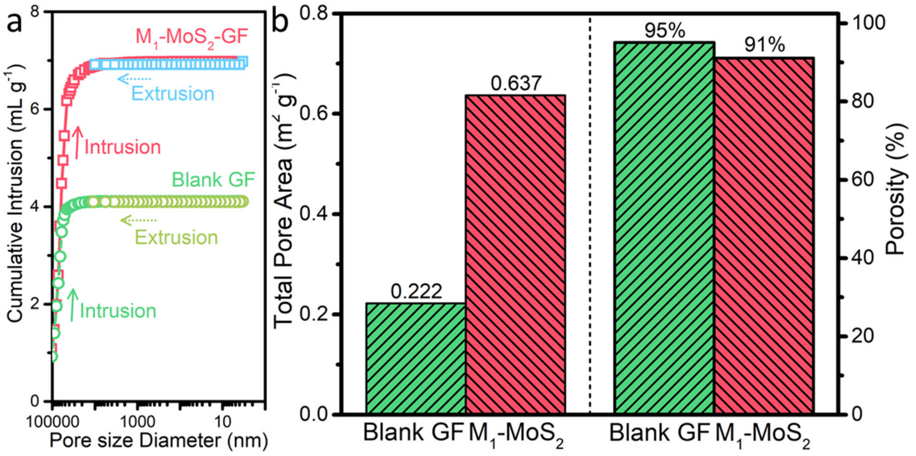

Supplementary Figure 8. (a) Intrusion and extrusion curves of blank GF and M_1-MoS_2-GF (M = Co) in mercury intrusion porosimetry and (b) comparisons in total pore area and porosity.

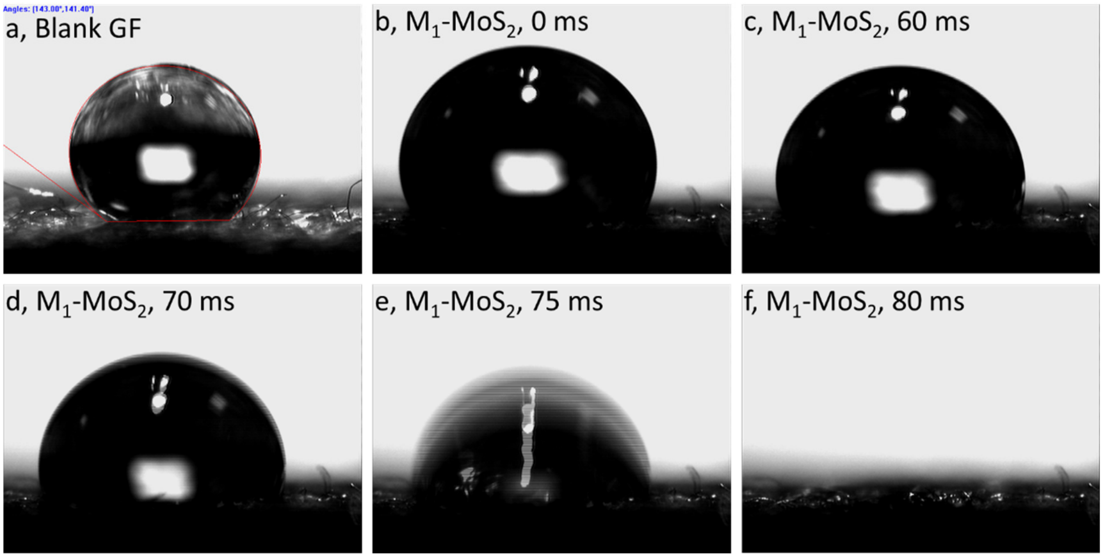

Supplementary Figure 9. (a) Water contact angle of blank GF and (b-f) M_1-MoS_2-GF (M = Co). Owing to its hydrophilicity and quick water absorption, we cannot determine an accurate value of contact angle for M_1-MoS_2-GF.

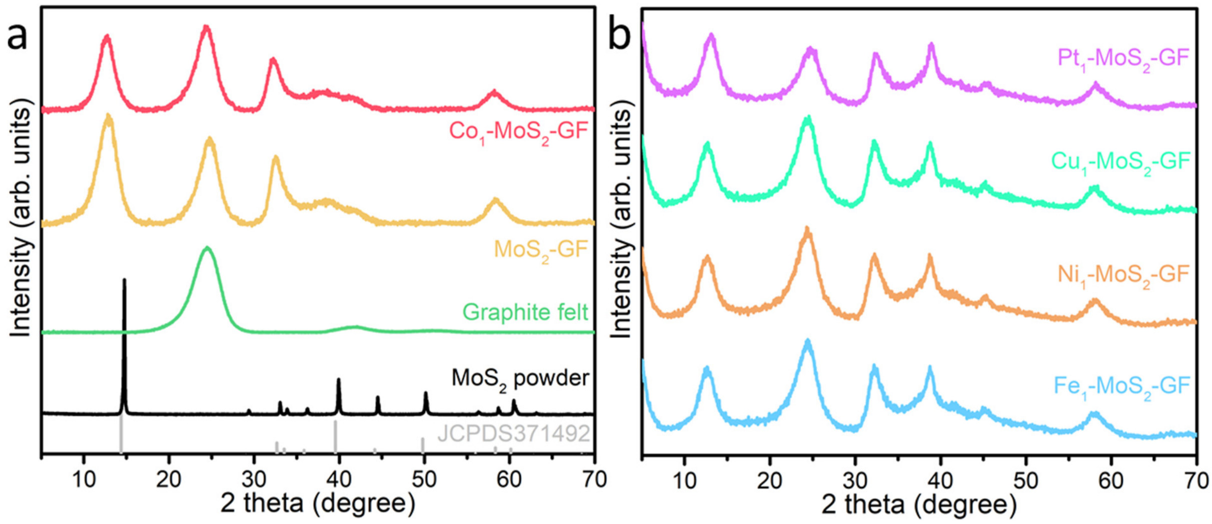

Supplementary Figure 10. (a) XRD patterns of MoS_2 powder, graphite felt, MoS_2-GF and Co_1-MoS_2-GF; (b) XRD patterns of Fe_1-MoS_2-GF, Ni_1-MoS_2-GF, Cu_1-MoS_2-GF and Pt_1-MoS_2-GF. The peak at ~ 39 º is attributed to the C {001}, which is sensitive to the position of graphite felt sample.

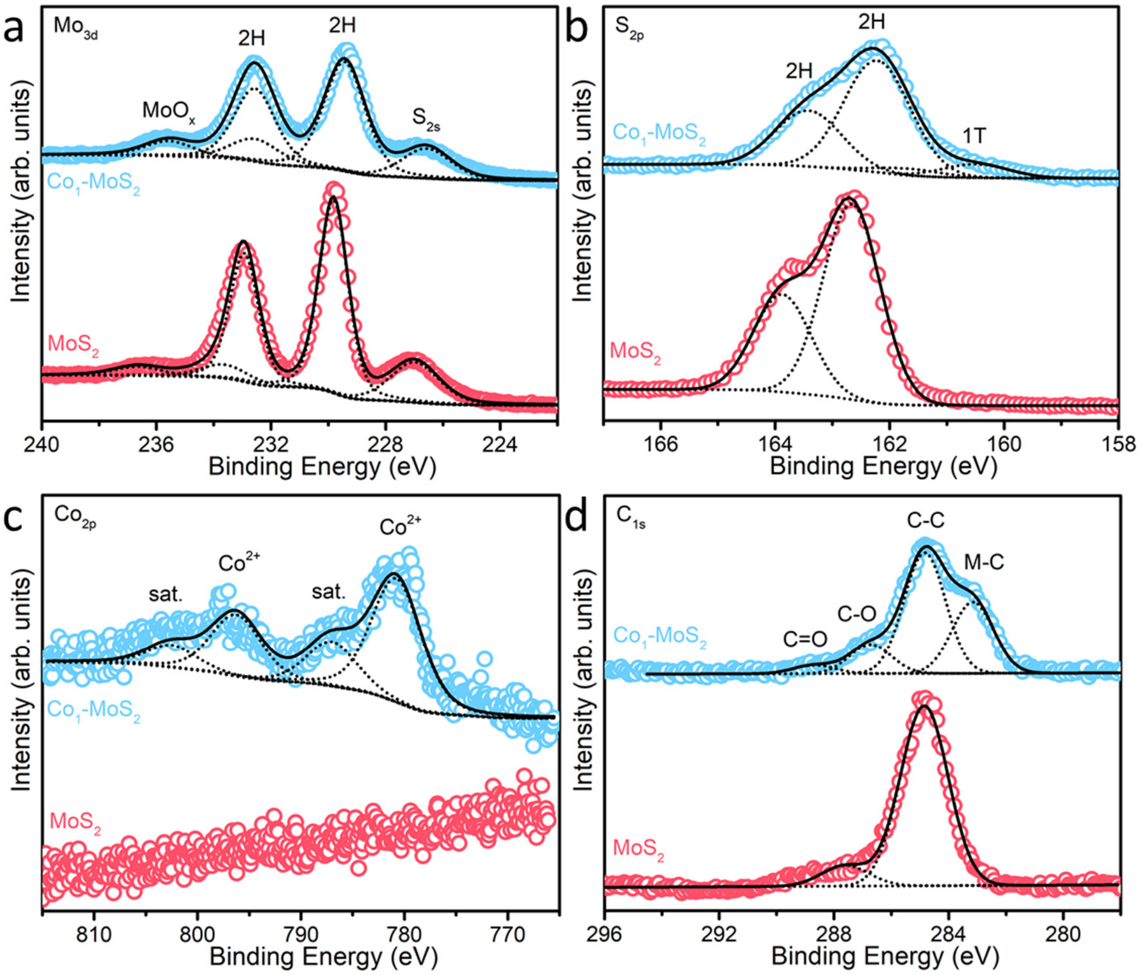

Supplementary Figure 11. (a) High resolution XPS Mo_{3d}, (b) S_{2p}, (c) Co_{2p} and (d) C_{1s} spectra of MoS_2-GF and Co_1-MoS_2-GF.

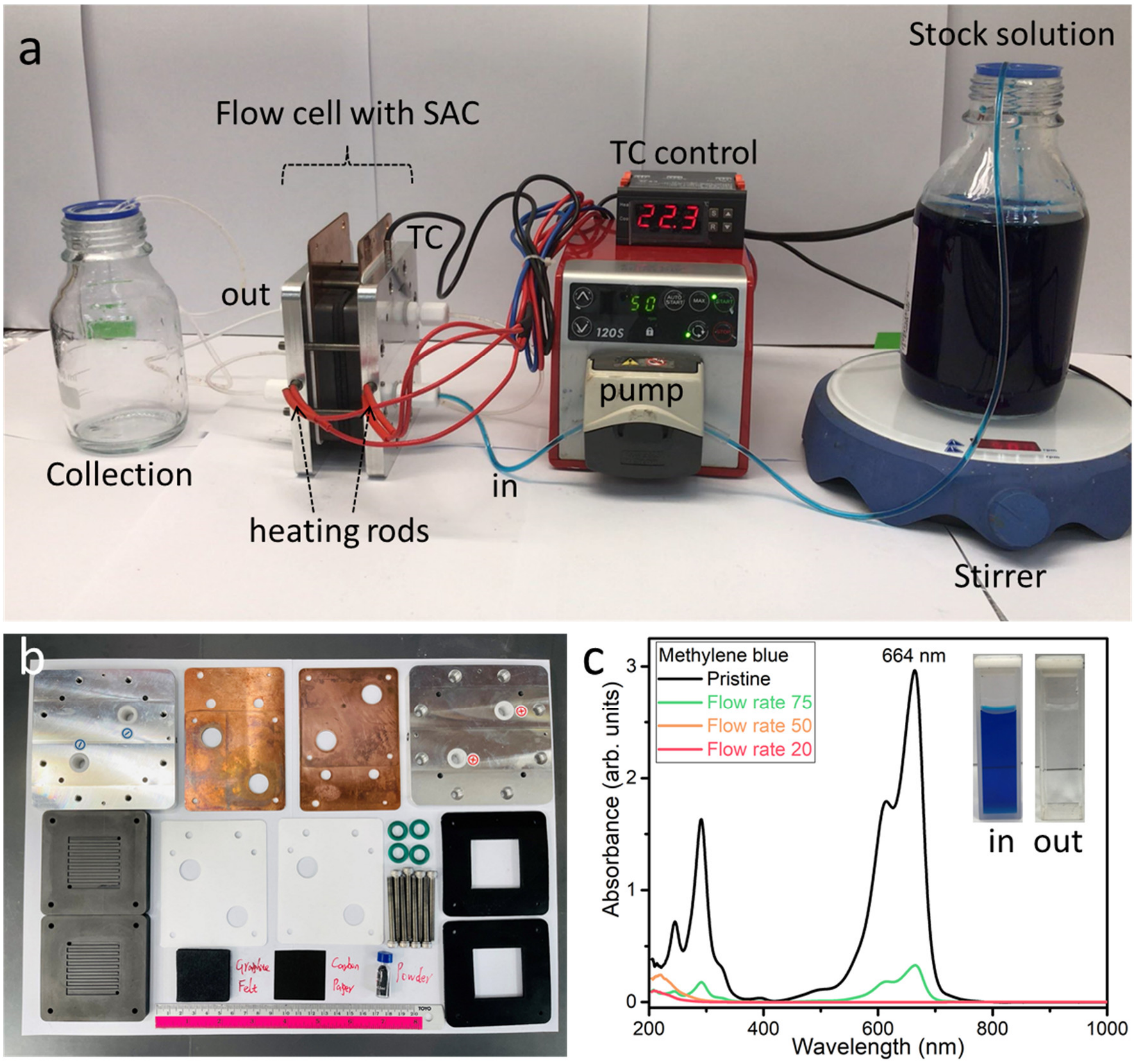

Supplementary Figure 12. Flow cell setup. (a) Photo of the flow cell setup with a peristaltic pump and a temperature controller for SAC-catalysed reactions; (b) components of the flow cell; (c) catalytic performance for the reduction of methylene blue by UV-Vis spectra. Inset shows the photos of methylene blue solution before and after decomposition. The flow reactor equipped with a Co_1-MoS_2-GF module exhibited > 99% efficiency for reductive decomposition of methylene blue and oxidative decomposition of rhodamine 6G (**Supplementary Fig. 13**) at suitable flow rates. Condition: 50 mg mL^{-1} methylene blue and 2 equiv. of $NaBH_4$ in ultrapure water at r. t.

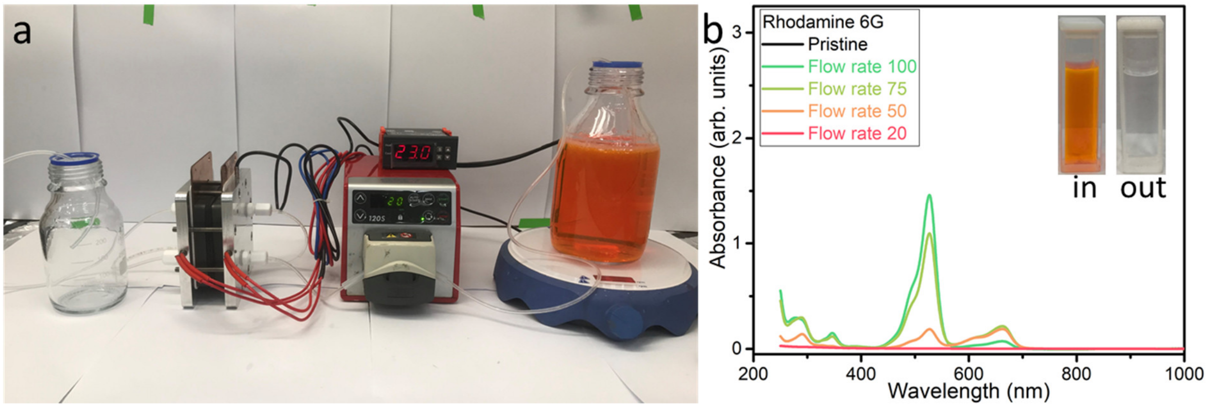

Supplementary Figure 13. (a) Flow cell setup for oxidative decomposition of rhodamine 6G using Co_1-MoS_2-GF; (b) UV-Vis spectra showing the catalytic efficiency at various flow rates. Inset shows the photos of rhodamine 6G solution before and after decomposition. Condition: 50 mg mL^{-1} rhodamine 6G and 50 equiv. of H_2O_2 in ultrapure water at r.t.

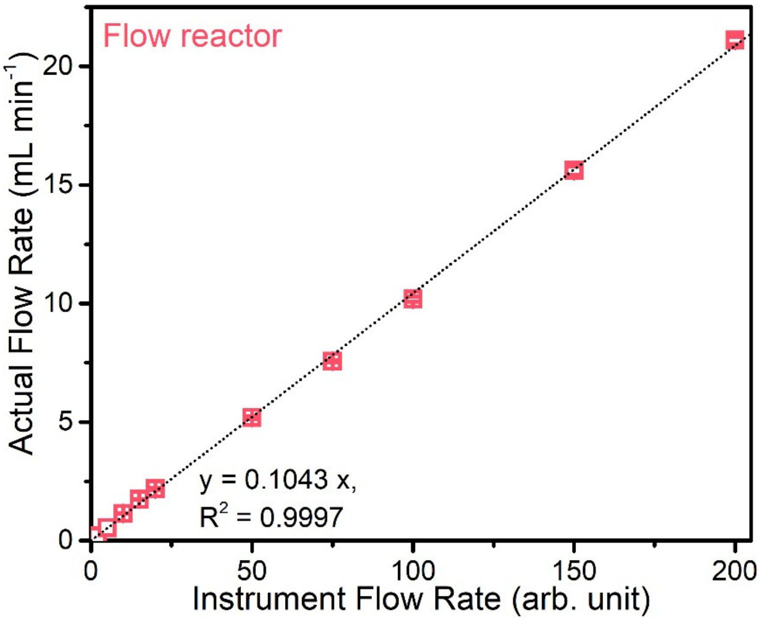

Supplementary Figure 14. Actual flow rate versus areal (instrument) flow rate by the peristaltic pump using Co_1-MoS_2-GF. Good linearity was observed in the whole range (1 ~ 200, equivalent to 0.1 ~ 20 mL min^{-1}) of flow reaction. Error bars (SD) were presented from 3 individual tests.

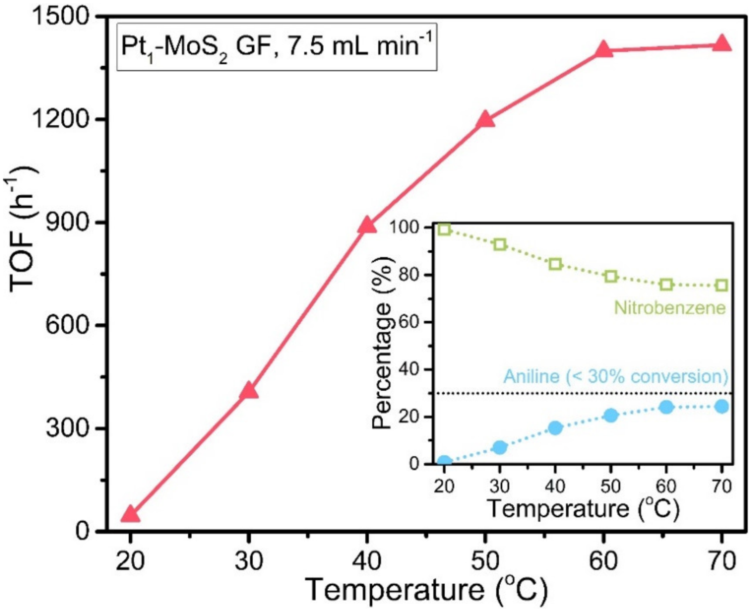

Supplementary Figure 15. Turnover frequency (TOF) analysis of Pt$_1$-MoS$_2$ in the reaction-limited regime. Inset shows the percentage of reactant and product at various temperatures. Condition: 0.10 M nitrobenzene, 0.05 M ammonia borane in acetonitrile/H_2O mixture (5:1 v/v) at a flow rate of 7.5 mL min^{-1}.

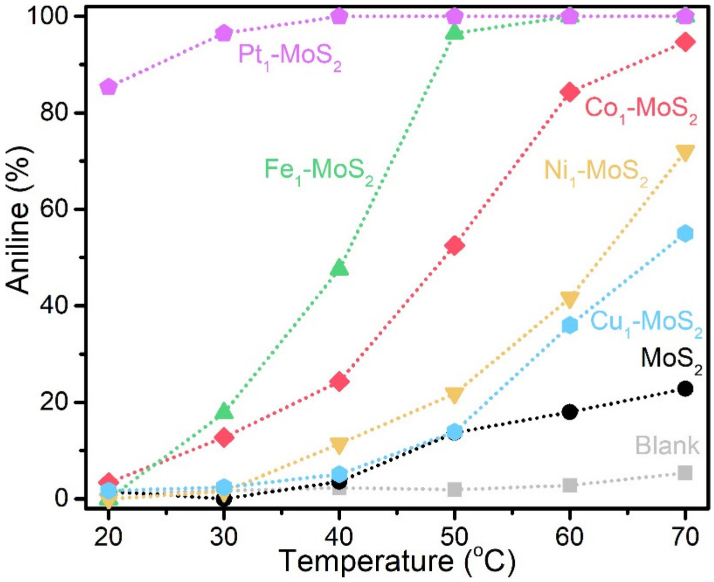

Supplementary Figure 16. Additional catalyst screening of various SACs and controls in the quantitative conversion regime. Condition: 0.025 M nitrobenzene with 0.050 M ammonia borane in acetonitrile/H_2O mixture (5:1, v/v) at a flow rate of 1 mL min^{-1}.

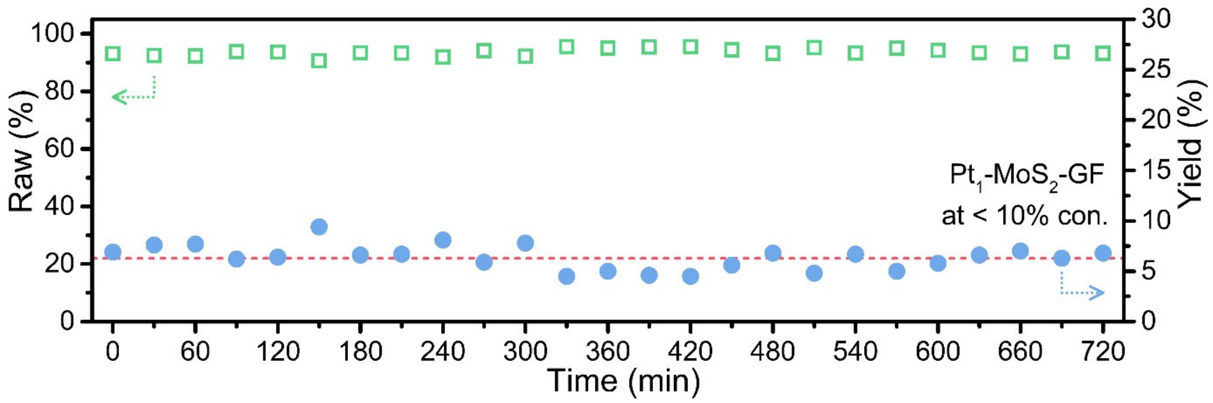

Supplementary Figure 17. Additional 12 h stability test of the Pt_1-MoS_2-GF catalyst at < 10% conversion. Condition: 0.20 M nitrobenzene with 0.05 M ammonia borane in acetonitrile/H_2O mixture (5:1, v/v) at a flow rate of 1 mL min^{-1}.

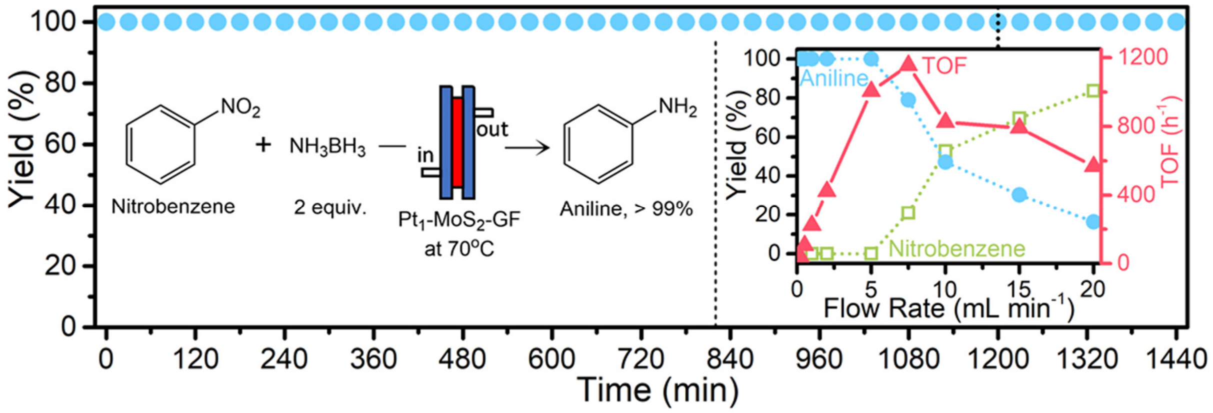

Supplementary Figure 18. A 24 h on-stream demonstration of the Pt_1-MoS_2-GF catalyst in the quantitative conversion regime. The yield versus flow rate test shown in the inset was performed at 20 h. Condition: 0.025 M nitrobenzene with 0.050 M ammonia borane in acetonitrile/H_2O mixture (5:1, v/v) at a flow rate of 1 mL min^{-1}.

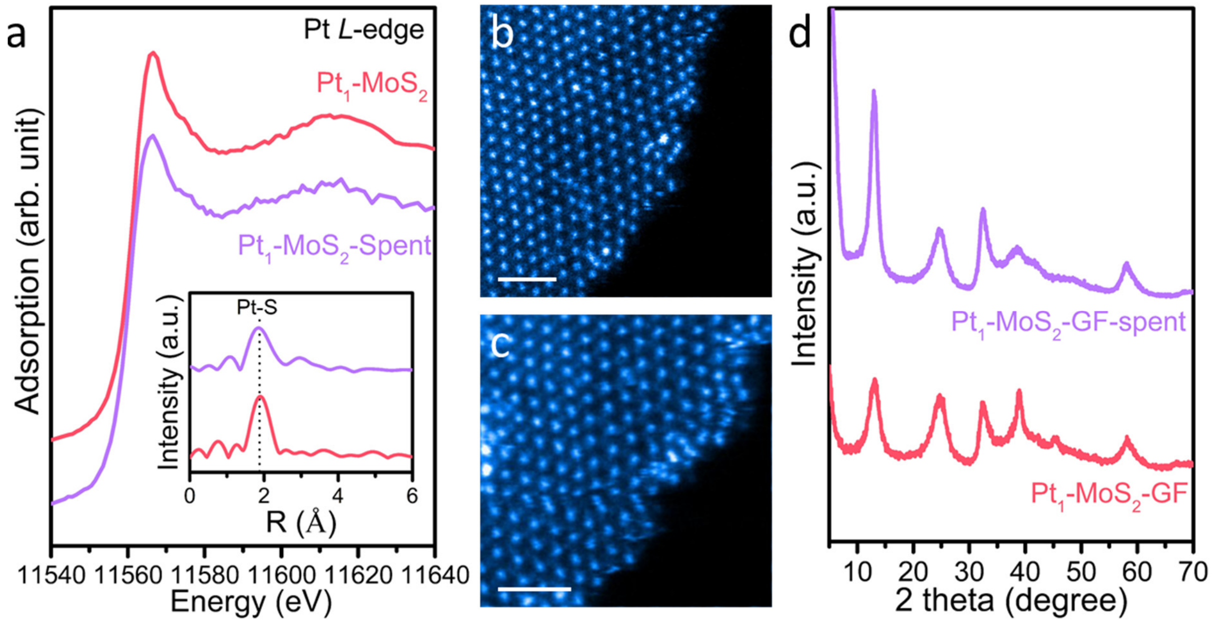

Supplementary Figure 19. Characterization of the spent Pt_1-MoS_2 catalyst. (a) XANES Pt L_3-edge spectra, inset shows the corresponding FT-EXAFS spectra; (b, c) STEM images of the spent catalyst; (d) XRD patterns. Scale bar: 1 nm.

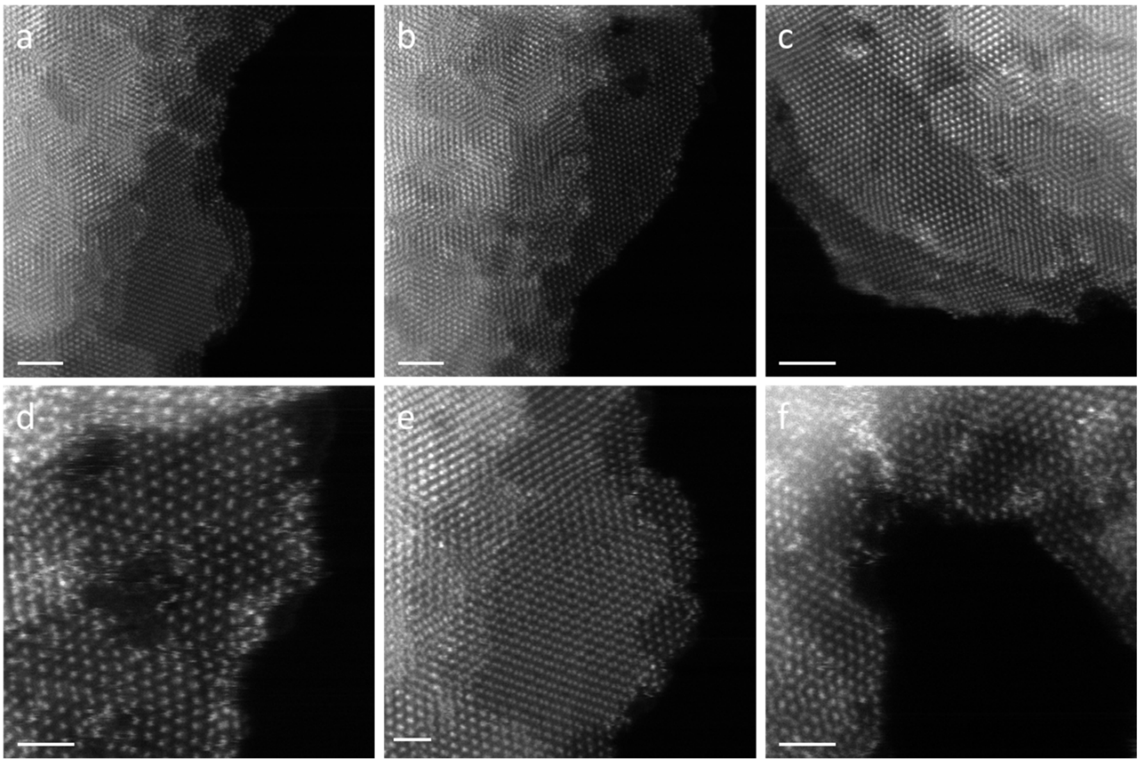

Supplementary Figure 20. (a-f) Representative STEM images of the spent Pt_1-MoS_2-GF catalyst. Scale bars: a-c, 2 nm; d-f, 1 nm.

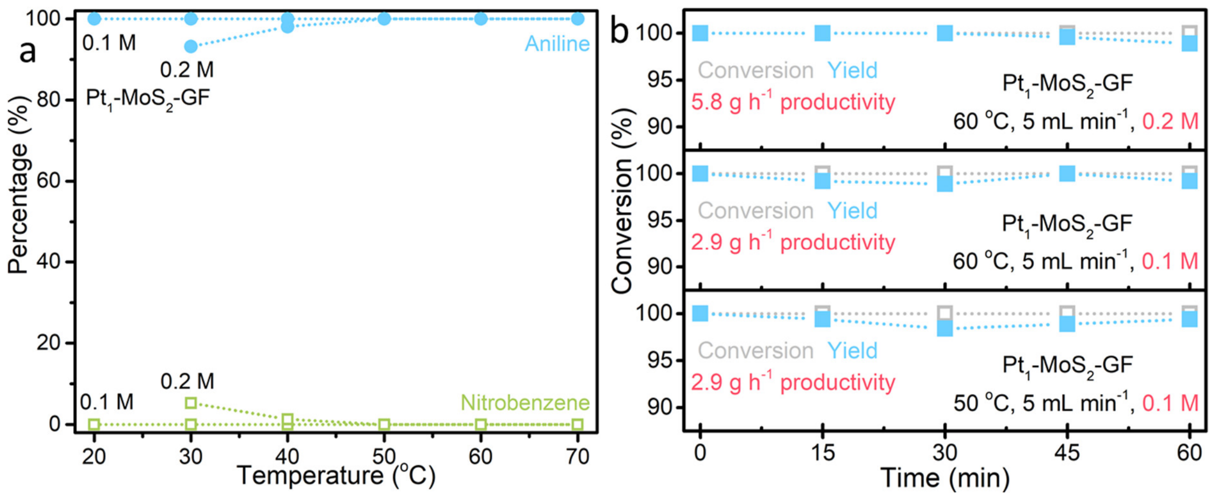

Supplementary Figure 21. Examination on flow reactor productivity at high concentration of reactant. (a) Temperature dependence of Pt_1-MoS_2-GF for nitrobenzene reduction at 0.1 M and 0.2 M of nitrobenzene; (b) Demonstration on one-hour continuous production in various conditions. Condition: 0.1 M nitrobenzene, 0.2 M ammonia borane in acetonitrile/H_2O mixture (1:1 v/v), or 0.2 M nitrobenzene, 0.4 M ammonia borane in acetonitrile/H_2O mixture (3:1 v/v) with 1 piece of Pt_1-MoS_2-GF (4 × 4 cm^2). Flow rate is fixed at 1 mL min^{-1} for temperature screening and 5 mL min^{-1} for productivity demonstration. The demonstration is performed at a reduced temperature compared to the stability test at 0.025 M in consideration of large amount of hydrogen gas produced. > 98% of GC yield was observed throughout the productivity test.

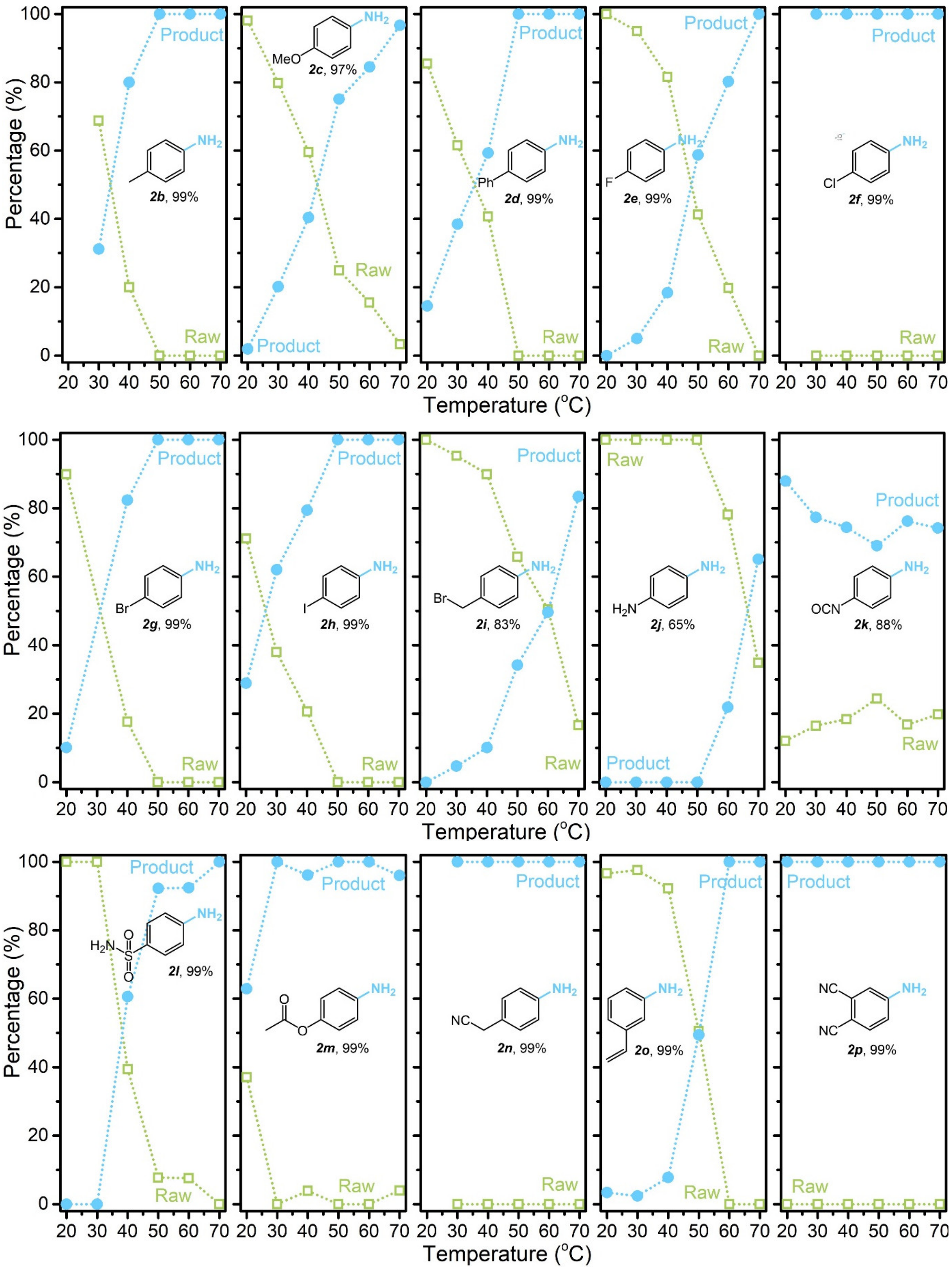

Supplementary Figure 22. Reaction profiles of various substrates using Pt_1-MoS_2-GF. Condition: 0.025 M nitroarenes, 0.050 M ammonia borane in acetonitrile/H_2O mixture (5:1, v/v) with 1 piece of Pt_1-MoS_2-GF (4×4 cm^2) at a flow rate of 1 mL min^{-1}. Inset shows the corresponding structure. 5% of DMF is added for those substrates with poor solubility in acetonitrile/H_2O mixture.

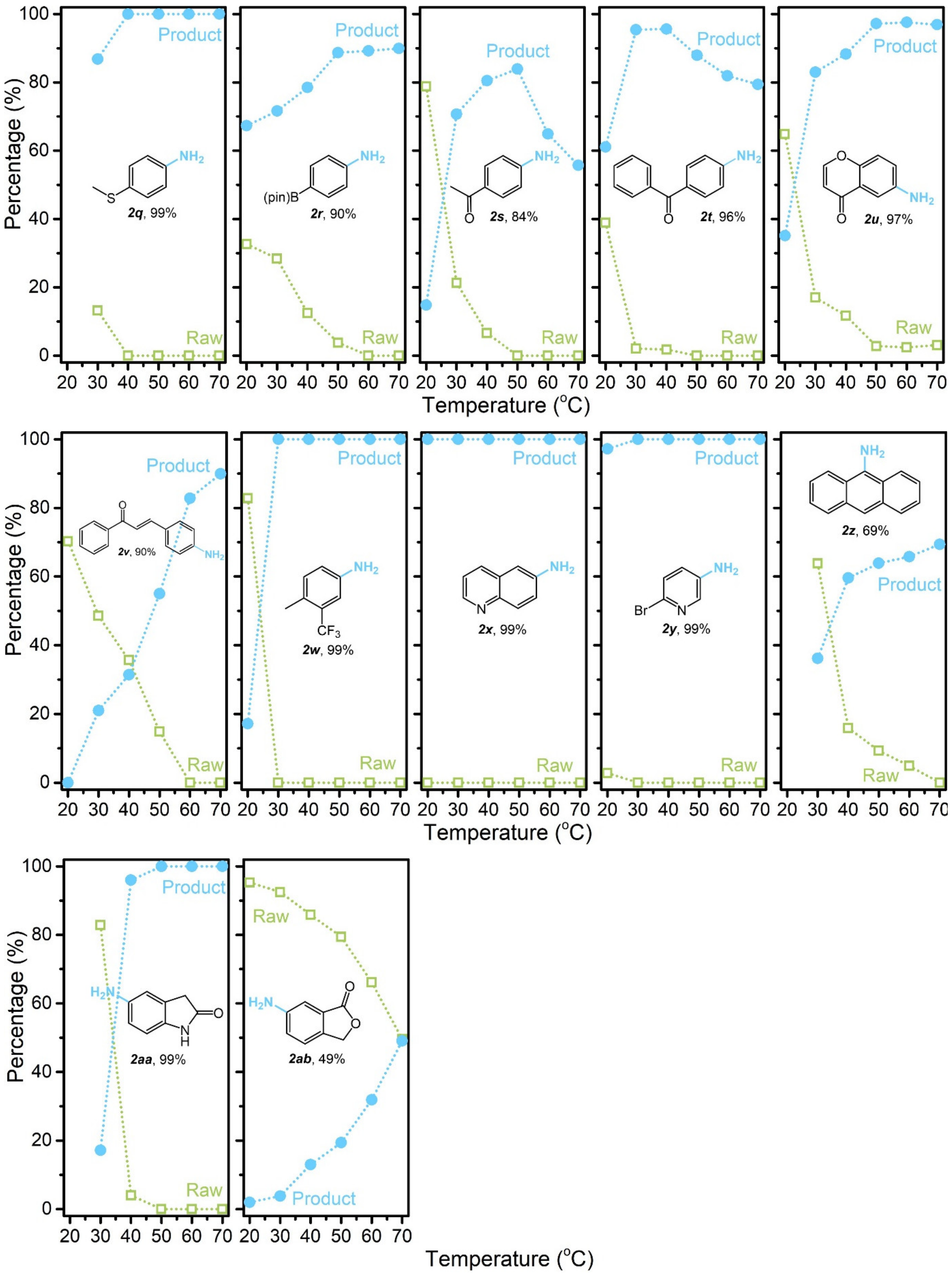

Supplementary Figure 22 (Continued). Reaction profiles of various substrates using Pt_1-MoS_2-GF. Condition: 0.025 M nitroarenes, 0.050 M ammonia borane in acetonitrile/H_2O mixture (5:1, v/v) with 1 piece of Pt_1-MoS_2-GF (4 × 4 cm^2) at a flow rate of 1 mL min^{-1}. Inset shows the corresponding structure. 5% of DMF is added for those substrates with poor solubility in acetonitrile/H_2O mixture.

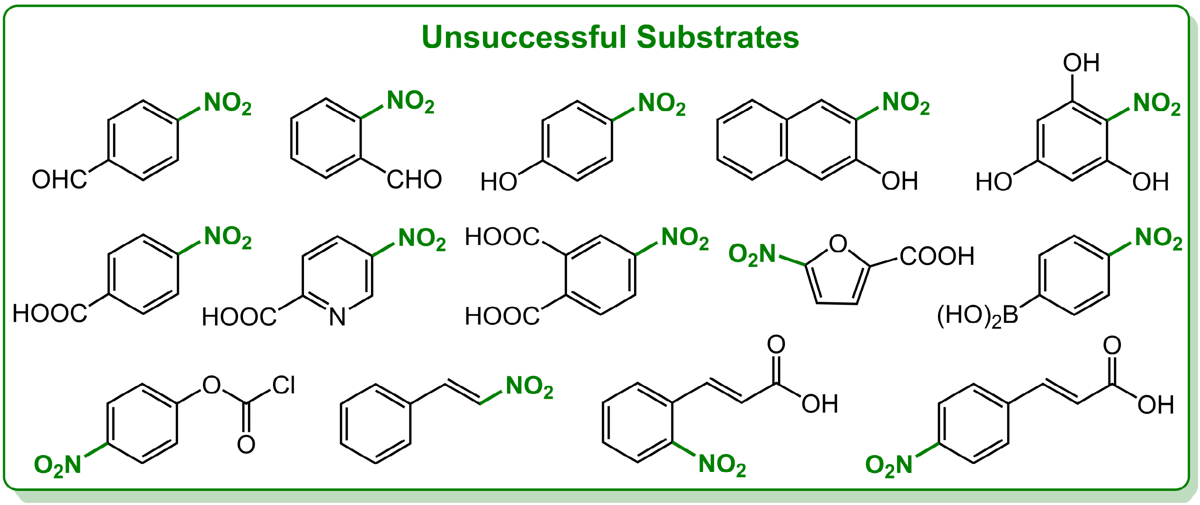

Supplementary Figure 23. Extended scope of unsuccessful substrates for the chemoselective nitroarene reduction using SAC-catalysed flow setup.

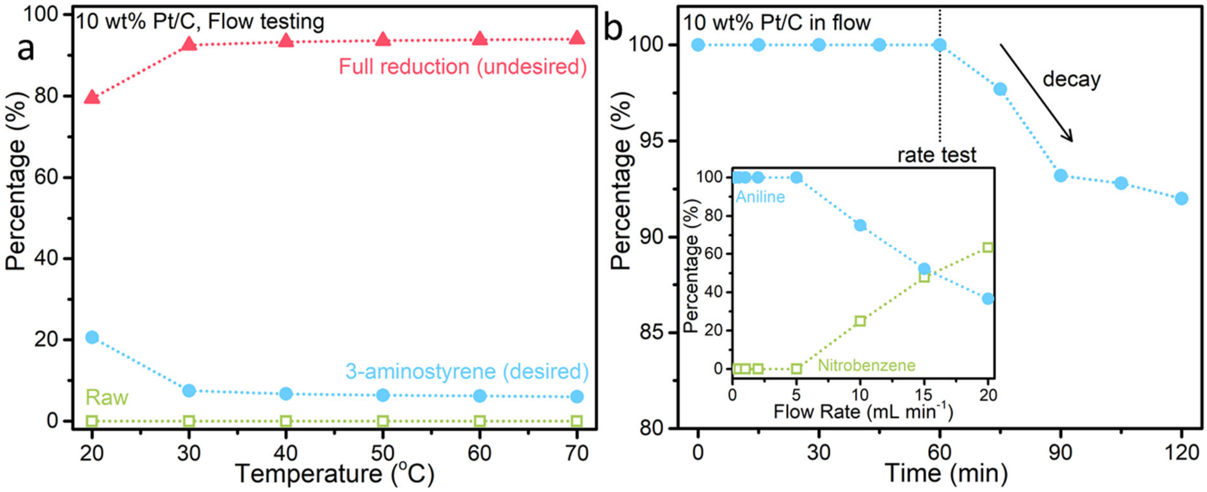

Supplementary Figure 24. (a) Catalytic performance and (b) stability testing of 10 wt% Pt/C for 3-nitrostyrene reduction in the flow reactor. Pt loading on graphite felt is kept to be the same as Pt_1-MoS_2-GF.

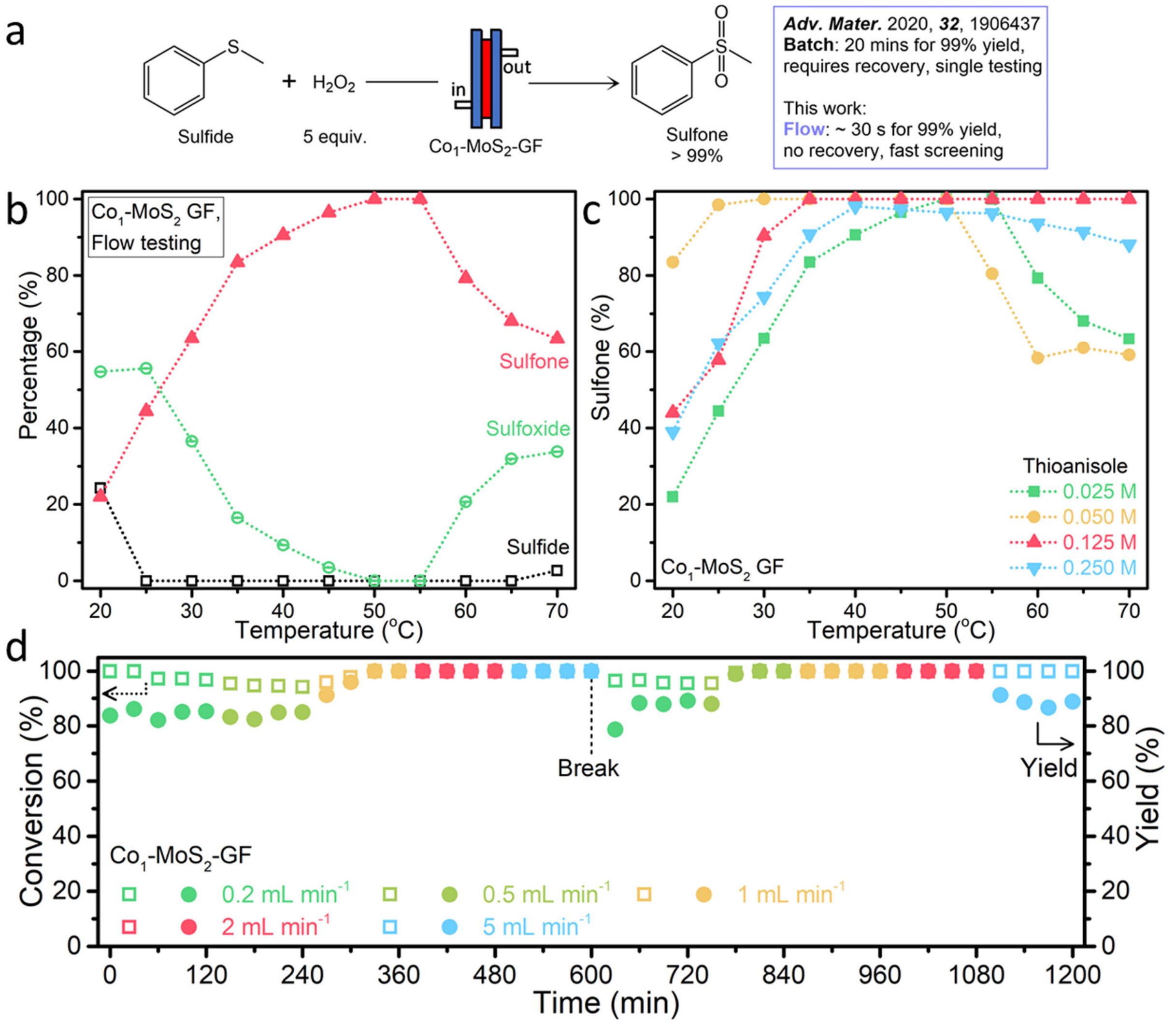

Supplementary Figure 25. Thioanisole oxidation by Co_1-MoS_2-GF in the flow setup. (a) Comparison of SAC-catalysed sulfide oxidation in batch and flow setup; (b) Catalytic performance using the Co_1-MoS_2-GF catalyst; (c) Influence of the concentration of thioanisole on catalytic performance; (d) A 20 h on-stream demonstration of the flow setup at various flow rates. Semi-oxidation product is detected at a low flow rate of 0.2 or 0.5 mL min^{-1} owing to the weaker interaction in laminar flow field at low flow rates.

In this study, we chose the chemoselective oxidation of sulfides as a model reaction to examine the productivity of the SAC reactors in continuous flow production. We have previously demonstrated a similar operation in batch for SAC-mediated late-functionalisation of Tamiflu. As shown in **Supplementary Fig. 25**, the oxidised product (sulfone) was obtained at a conversion rate and yield of > 99%. The reaction was carried out at 50 °C with a short retention time of approximately 30 s and a flow rate of 2 mL min^{-1}. This reaction is significantly faster than the previous batch operation (~ 20 min). Even when the concentration is increased by 10-fold (0.25 M thioanisole), complete conversion and yield can be obtained. This is equivalent to a productivity of 4.7 g of isolated product (methyl phenyl sulfone) per hour, and it can be further increased by increasing the flow rate. We also performed a 20 h on-stream demonstration of the flow cell reactor (**Supplementary Fig. 25d**), which shows a highly reproducible performance at various flow rates (0.2 ~ 5 mL min^{-1}). This indicates that catalyst leaching and deactivation were minimal.

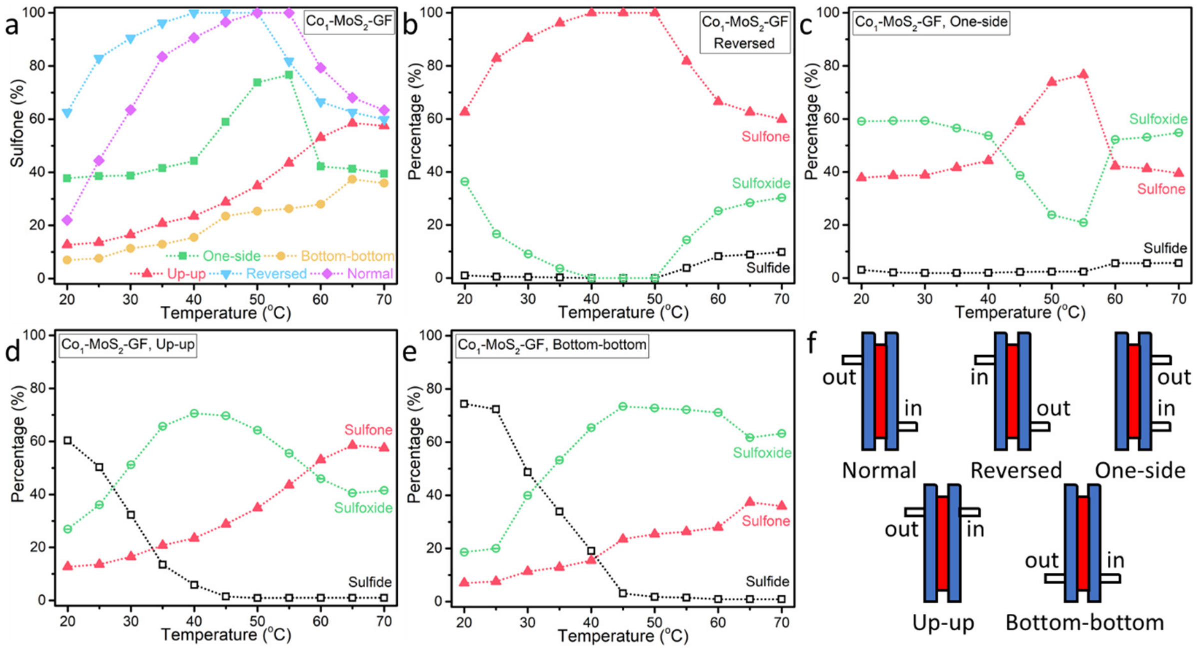

Supplementary Figure 26. (a) Catalytic performance of various flow cell configurations (f) for thioanisole oxidation using Co_1-MoS_2-GF; (b) Reversed configuration; (c) One-side configuration; (d) Up-up configuration and (e) Bottom-bottom configuration. Condition: 0.025 M thioanisole with 0.125 M H_2O_2 in acetonitrile at a flow rate of 2 mL min^{-1}.

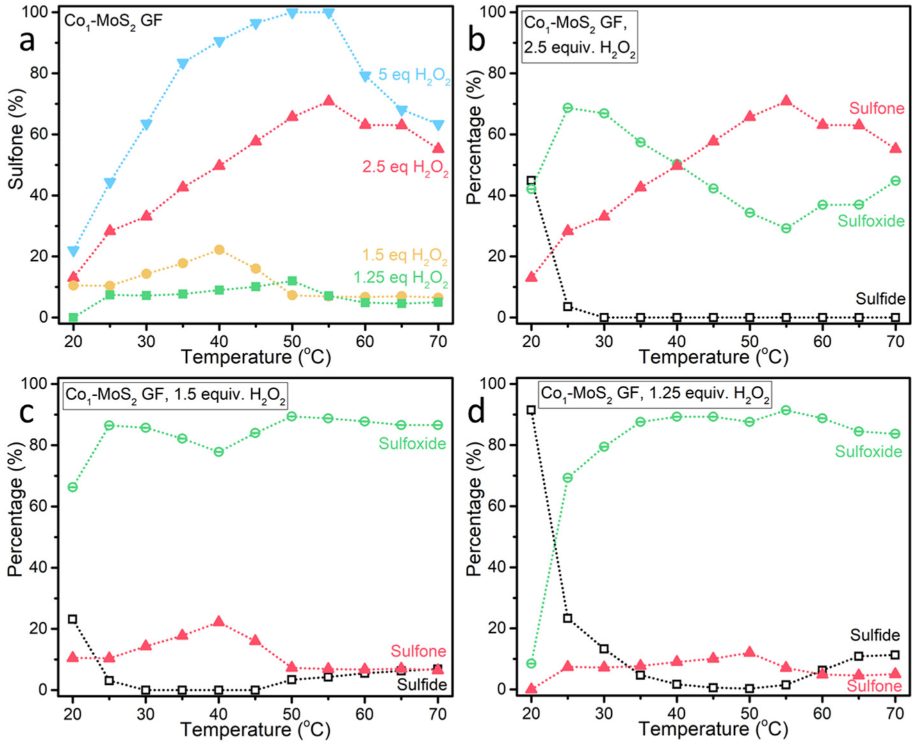

Supplementary Figure 27. (a) Influence of the amount of H_2O_2 on catalytic oxidation of thioanisole using Co_1-MoS_2-GF; (b) 2.5 equiv. of H_2O_2 (0.0625 M); (c) 1.5 equiv. of H_2O_2 (0.0375 M); (d) 1.25 equiv. of H_2O_2 (0.0313 M). Condition: 0.025 M thioanisole with various amounts of H_2O_2 in acetonitrile at a flow rate of 2 mL min^{-1}.

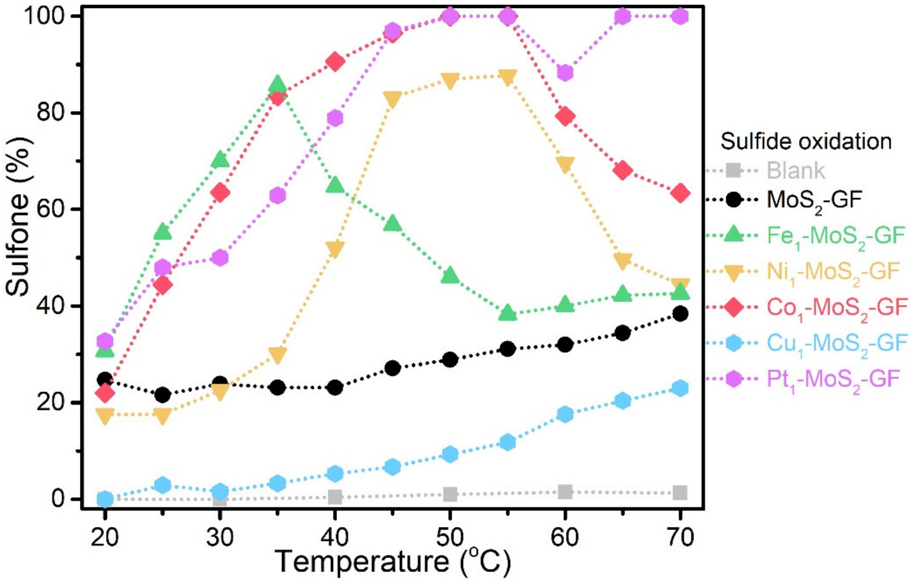

Supplementary Figure 28. Comparison in thioanisole oxidation using various catalysts. Condition: 0.025 M thioanisole with 0.125 M H_2O_2 in acetonitrile at a flow rate of 2 mL min^{-1}.

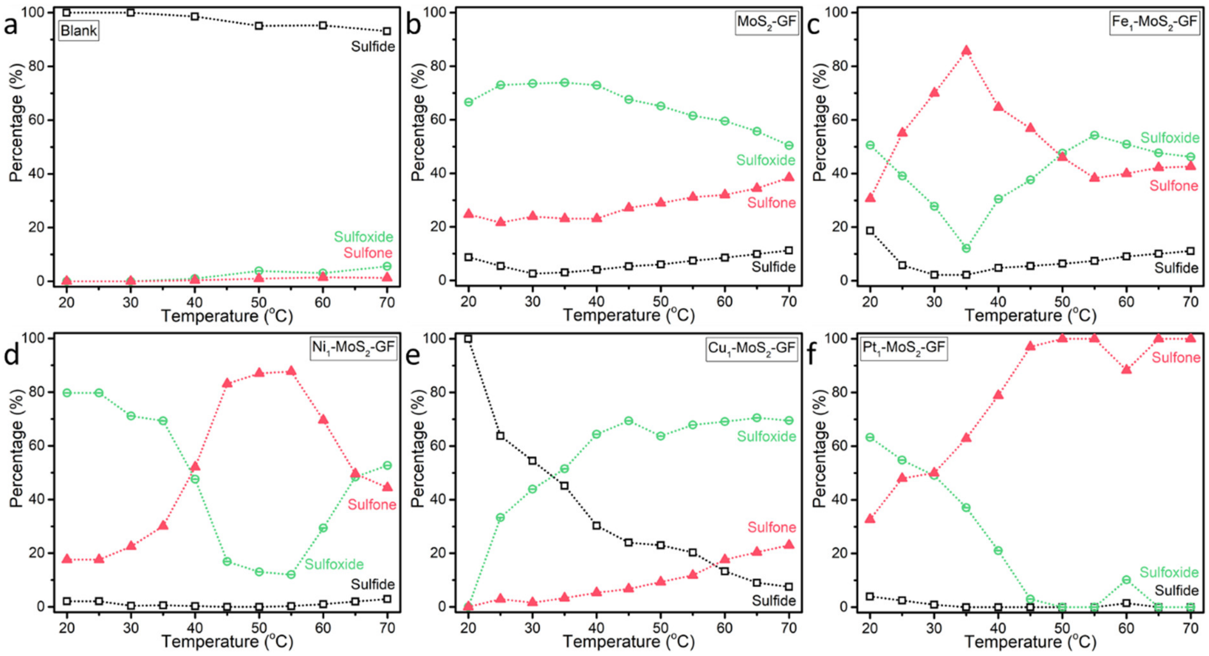

Supplementary Figure 29. (a) Catalytic performance for thioanisole oxidation using blank GF; (b) MoS_2-GF; (c) Fe_1-MoS_2-GF; (d) Ni_1-MoS_2-GF; (e) Cu_1-MoS_2-GF and (f) Pt_1-MoS_2-GF. Condition: 0.025 M thioanisole with 0.125 M H_2O_2 in acetonitrile at a flow rate of 2 mL min^{-1}.

Owing to its inherent advantages of fast reaction kinetics and no catalyst recovery step, the SAC-flow reactor can be used for fast screening of reaction types, flow cell configuration, catalyst module and reagent ratio (H_2O_2 to thioanisole) (as shown in **Supplementary Fig. 26 ~ 29**), demonstrating the ease of process optimisation. For instance, > 85% selectivity to semi-oxidation product (sulfoxide) can be achieved with 1.25 equivalent of H_2O_2.

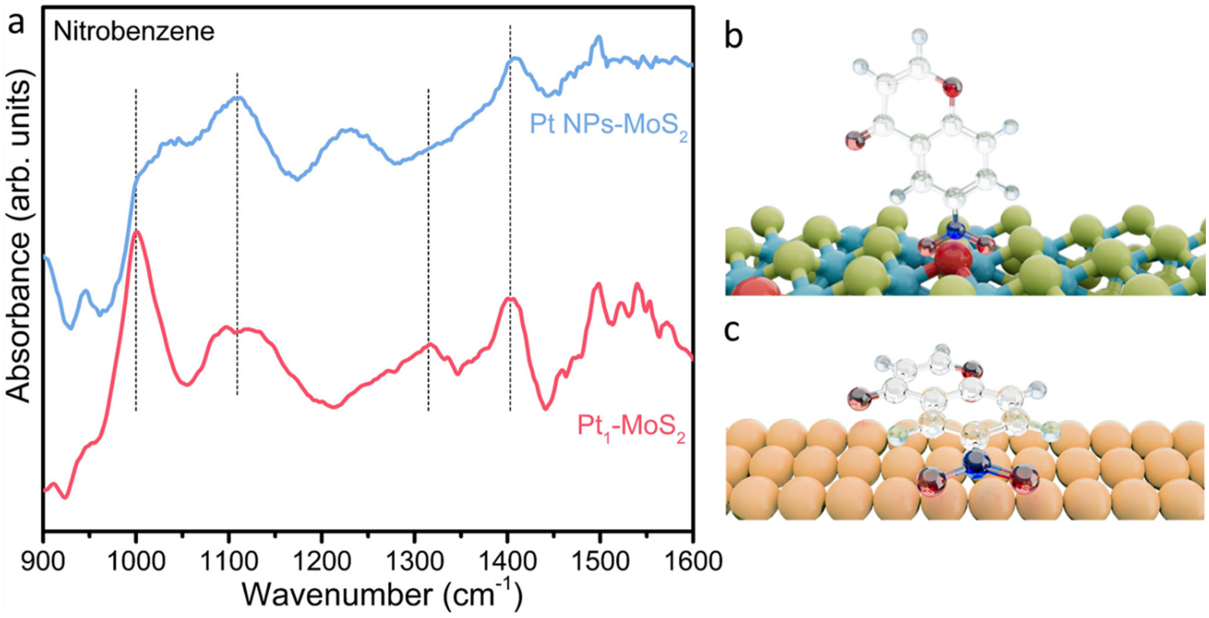

Supplementary Figure 30. (a) *In-situ* DRIFTS spectra of Pt_1-MoS_2 and Pt nanoparticles on MoS_2 toward 3-nitrostyrene hydrogenation at 70 °C; (b) Absorption mode of 3-nitrostyrene on Pt_1-MoS_2 (end-on configuration) and (c) Pt nanoparticles on MoS_2 (planar configuration), showing the origin of chemoselectivity.

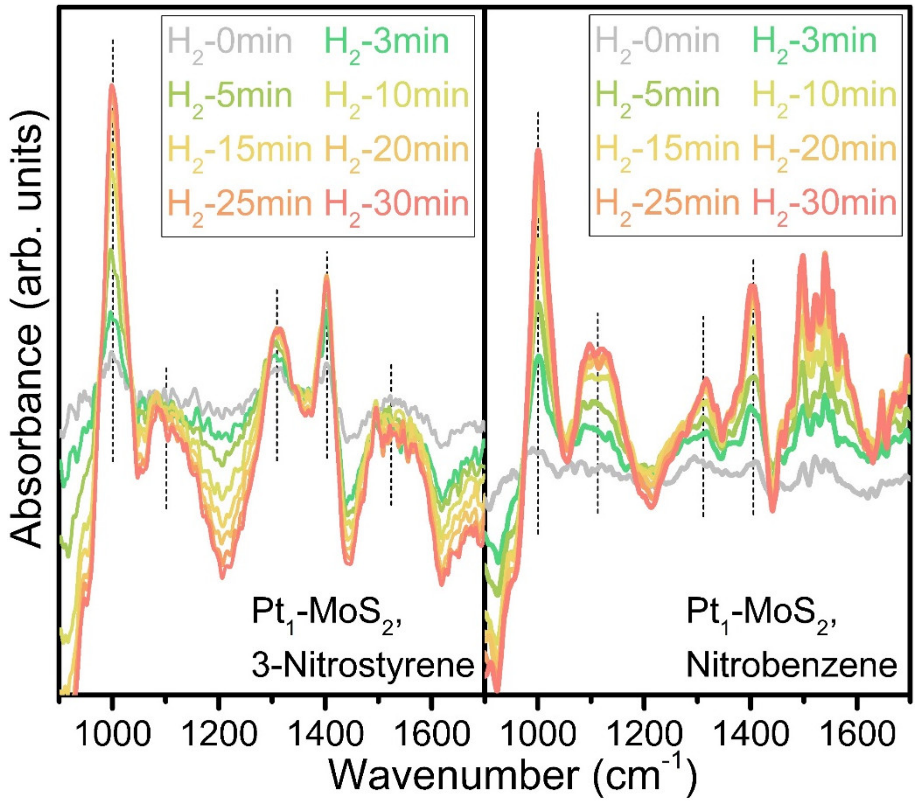

Supplementary Figure 31. *In-situ* DRIFTS spectra of Pt_1-MoS_2 toward 3-nitrostyrene and nitrobenzene hydrogenation at 70 °C at various reaction times.

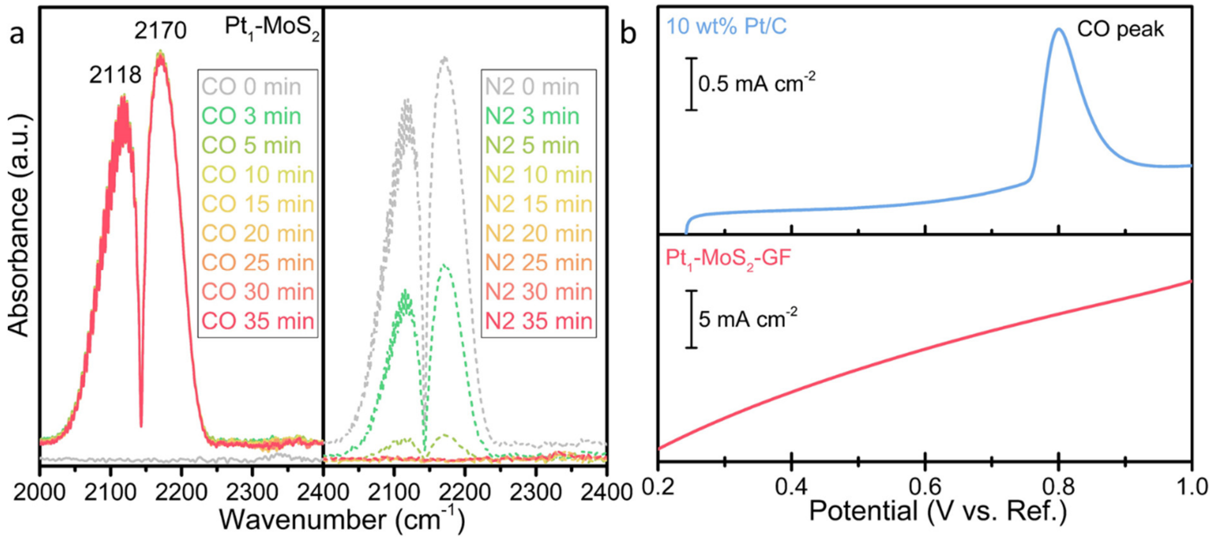

Supplementary Figure 32. (a) CO DRIFT experiment for Pt_1-MoS_2 at room temperature; (b) CO electrochemical stripping experiment for 10 wt% Pt/C and Pt_1-MoS_2-GF. No CO adsorption or stripping peak associated to nanoparticles can be observed in both cases.

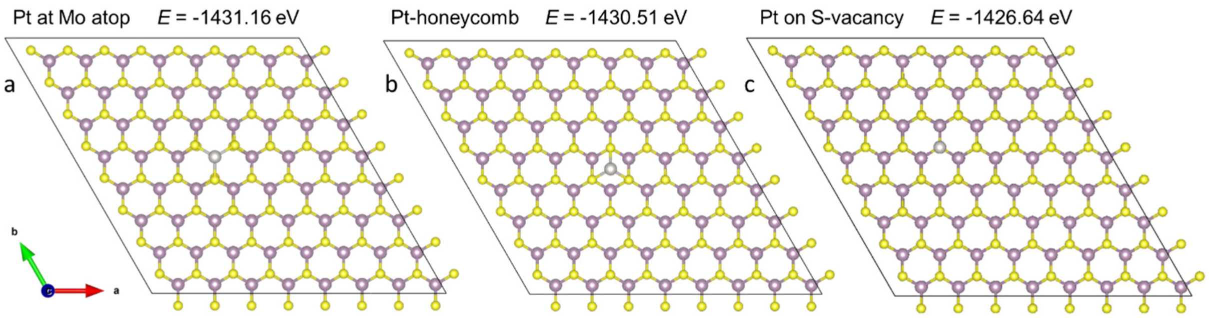

Supplementary Figure 33. Optimized configurations for (a) Pt SAC at the Mo atop site (dominated species in STEM, 93%), (b) Pt SAC on the honeycomb site (minor species, 7%) and (c) Pt SAC at the sulfur vacancy site (possible configuration). The Mo atop site is more stable from their formation energies without entropy (*E*). It is unlikely to obtain Pt SAC at the Mo vacancy (*i.e.* replacing Mo by Pt) in our wet impregnation method. The color scheme used: purple for Mo, yellow for S and white-grey for Pt.

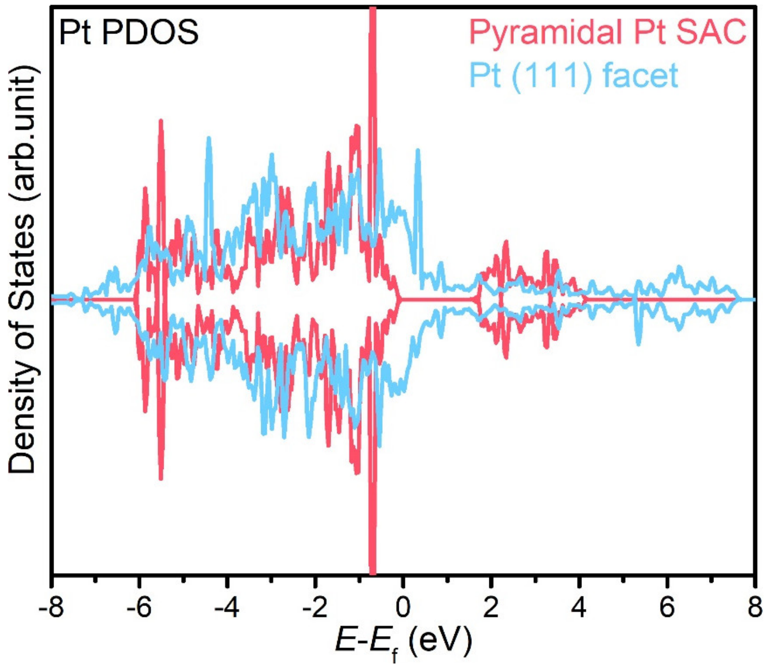

Supplementary Figure 34. Density of state (DOS) of Pt atom in the pyramidal Pt SAC at the Mo atop site and bulk Pt (111) surface. The *d*-band centers of them are -2.689 and -2.451 eV, respectively.

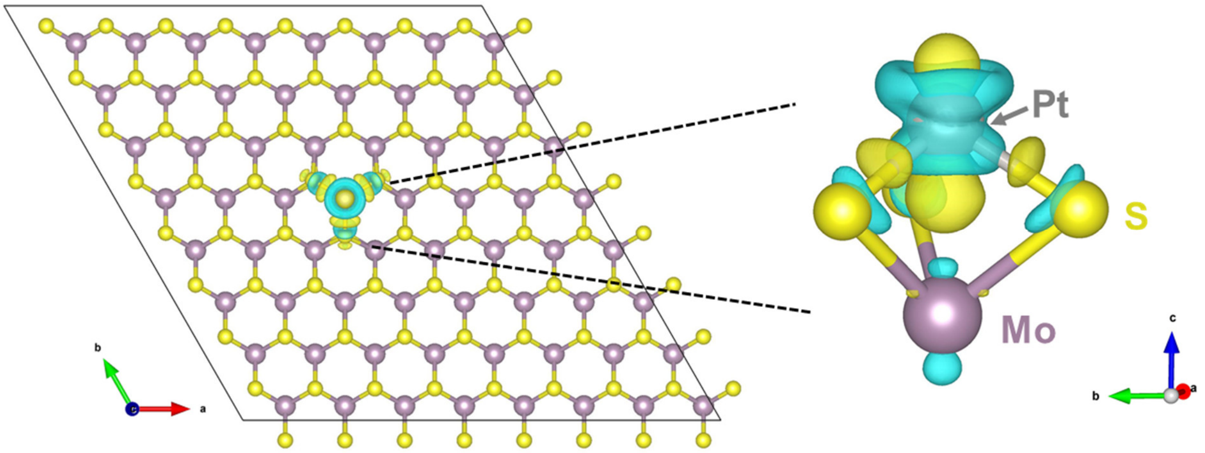

Supplementary Figure 35. Differential charge density of the pyramidal Pt SAC at the Mo atop site. Yellow and cyan regions in differential charge density represent electron accumulation and depletion. The Pt atom forms strong covalent bonds with nearby S atoms. Meanwhile, part of the charge is obtained from the Mo atom via the σ bond due to the higher electronegativity of Pt, making it electron-rich in the z direction. The color scheme used: purple for Mo, yellow for S and white-grey for Pt.

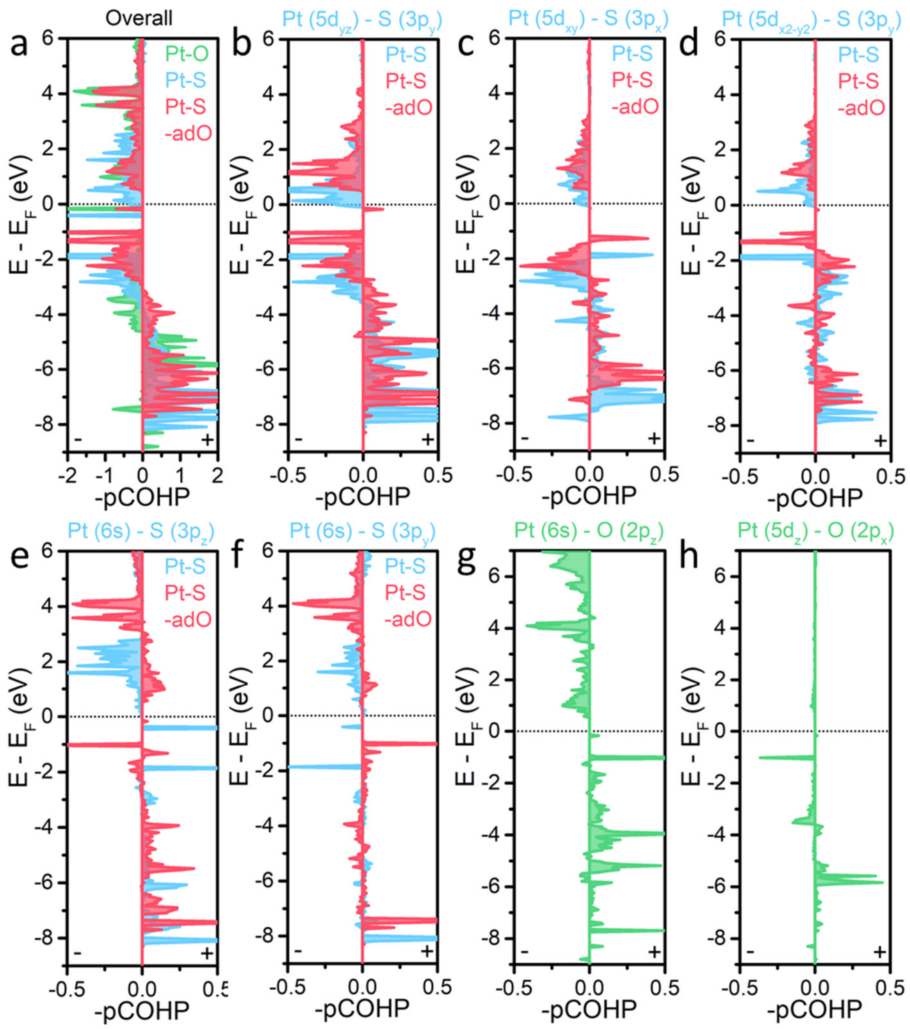

Supplementary Figure 36. The -pCOHP curves for 3-nitrostyrene adsorption on Pt-3S at the Mo atop site: (a) Comparison between the -pCOHP curves for Pt-O, Pt-S and Pt-S with nitro-adsorption; (b-f) Bonding orbitals for the Pt-S; (g, h) Bonding orbitals for the Pt-O.

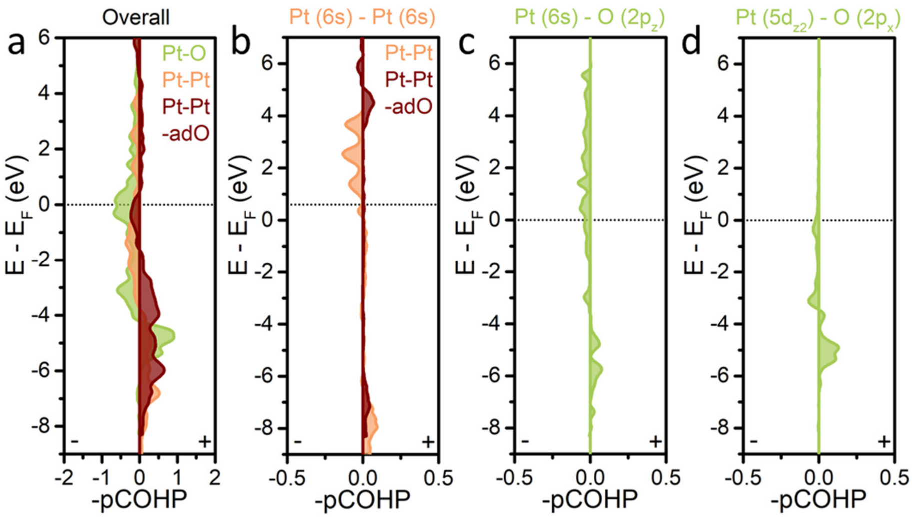

Supplementary Figure 37. The -pCOHP curves for 3-nitrostyrene adsorption on bulk Pt (111) facet: (a) Comparison between the -pCOHP curves for Pt-O, Pt-Pt and Pt-Pt with nitro-adsorption; (b) Representative bonding orbitals for the Pt-Pt; (c, d) Bonding orbitals for the Pt-O.

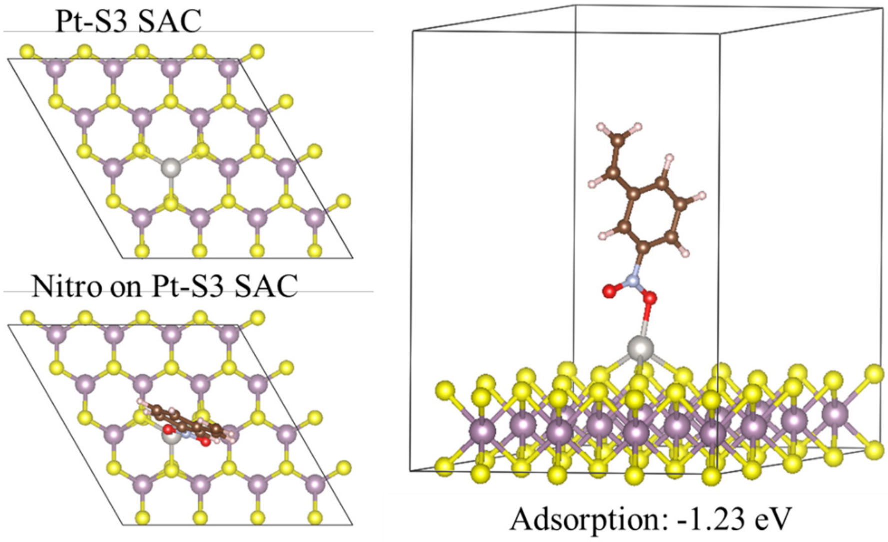

Supplementary Figure 38. The optimized end-on configuration of 3-nitrostyrene on Pt-3S at the Mo atop site. The color scheme used: purple for Mo, yellow for S, white-grey for Pt, red for O, light-blue for N, brown for C and white-pink for H.

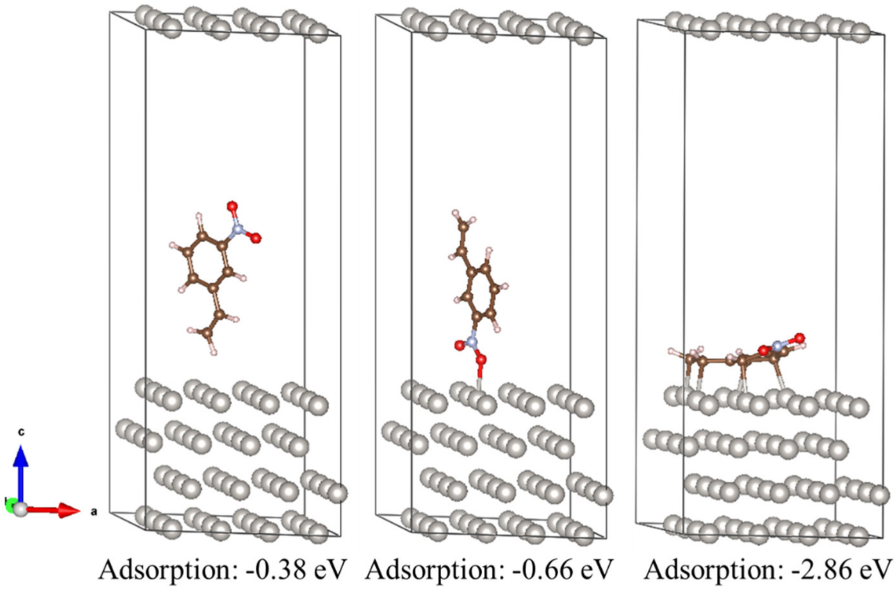

Supplementary Figure 39. The optimized configurations of 3-nitrostyrene on Pt (111) facet. The color scheme used: white-grey for Pt, red for O, light-blue for N, brown for C and white-pink for H.

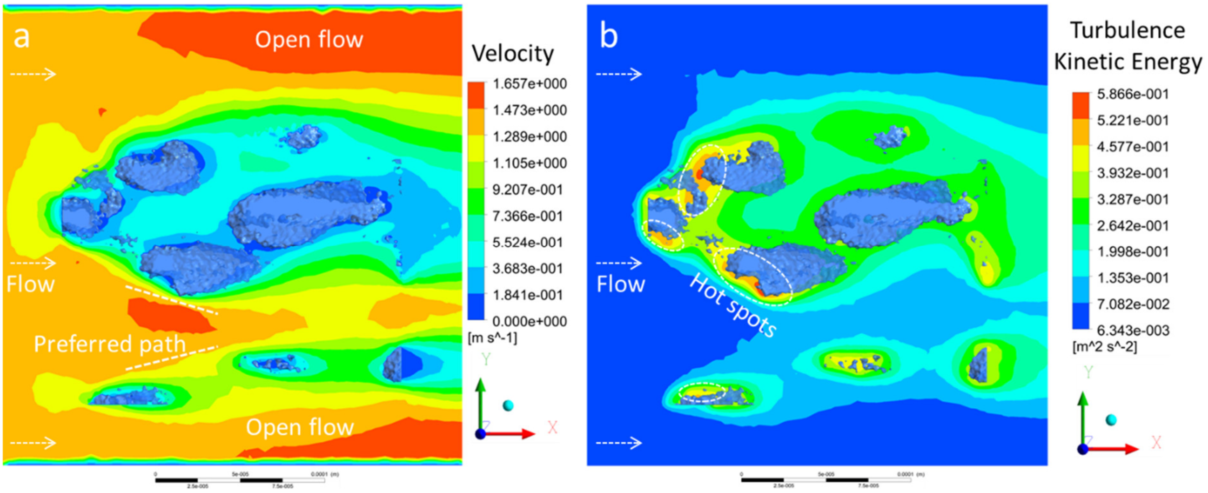

Supplementary Figure 40. Reactor-level understanding by CFD. (a) Velocity contour plot and (b) the corresponding turbulence kinetic energy contour plot at the XY plane for the fluidic behaviour of the catalyst model in the X direction from micro-CT; Fluidic hot spots and preferred paths are highlighted by open white cycles and arrows.

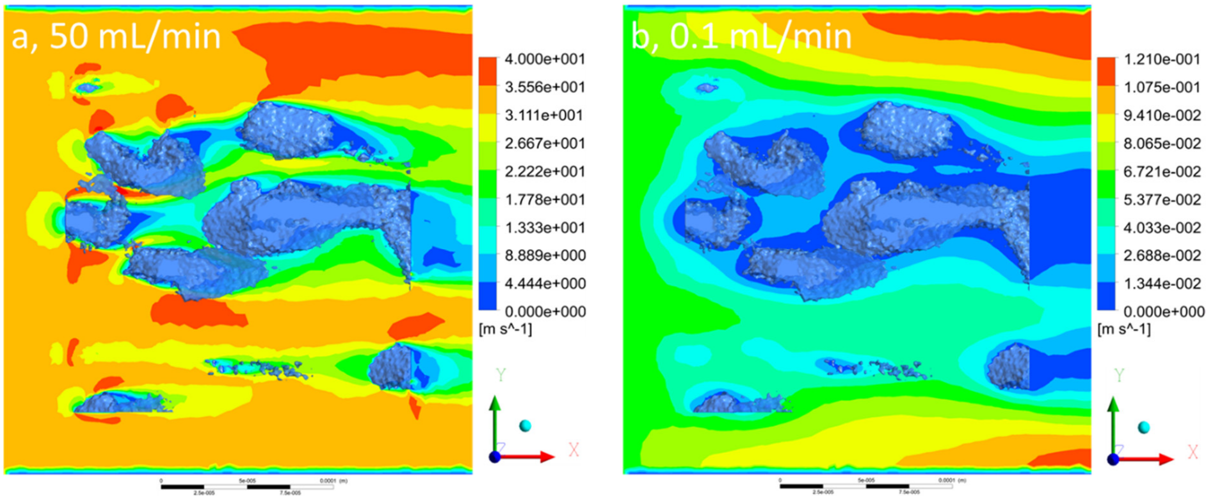

Supplementary Figure 41. Velocity contour at the XY plane for fluidic behavior at the **X** direction at a flow rate of (a) 50 mL min^{-1} and (b) 0.1 mL min^{-1}; Z height (+Z from the basal plane) is 55 μm.

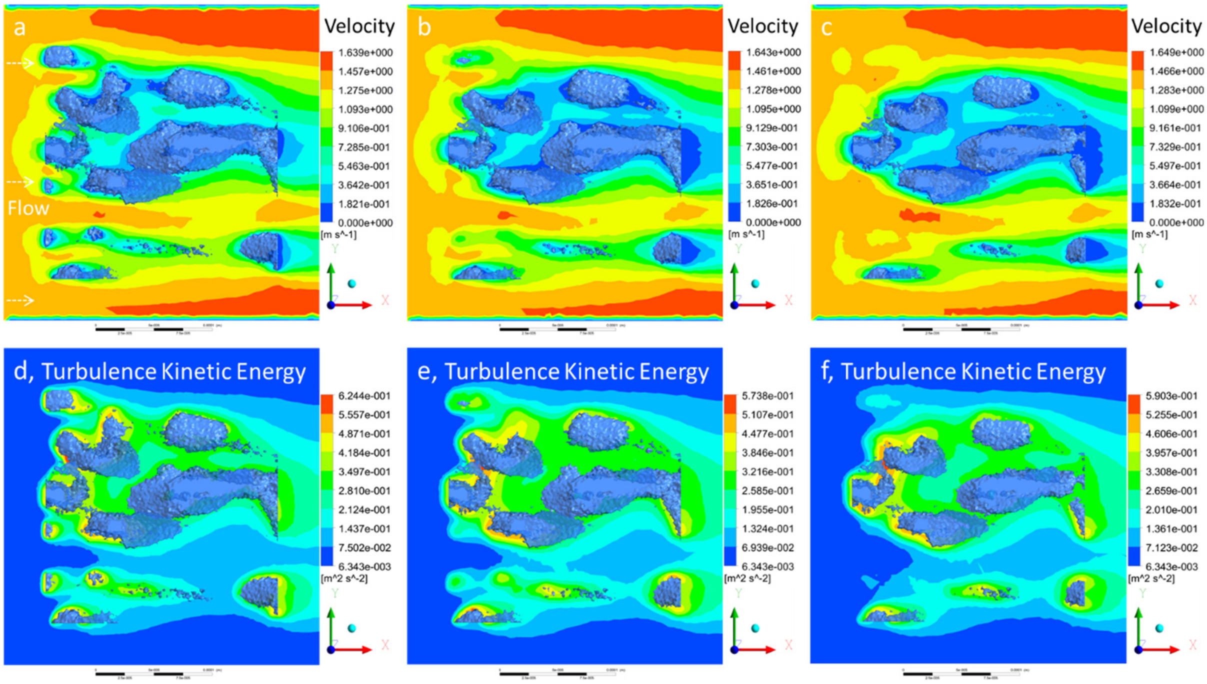

Supplementary Figure 42. (a-c) Velocity contour plots and (d-f) corresponding turbulence kinetic energy contour plots at the XY plane for fluidic behavior at the **X** direction; Z height (+Z from the basal plane) for a, b, c and the one showing in **Supplementary Fig. 40** are 40, 45, 50 and 55 µm, respectively.

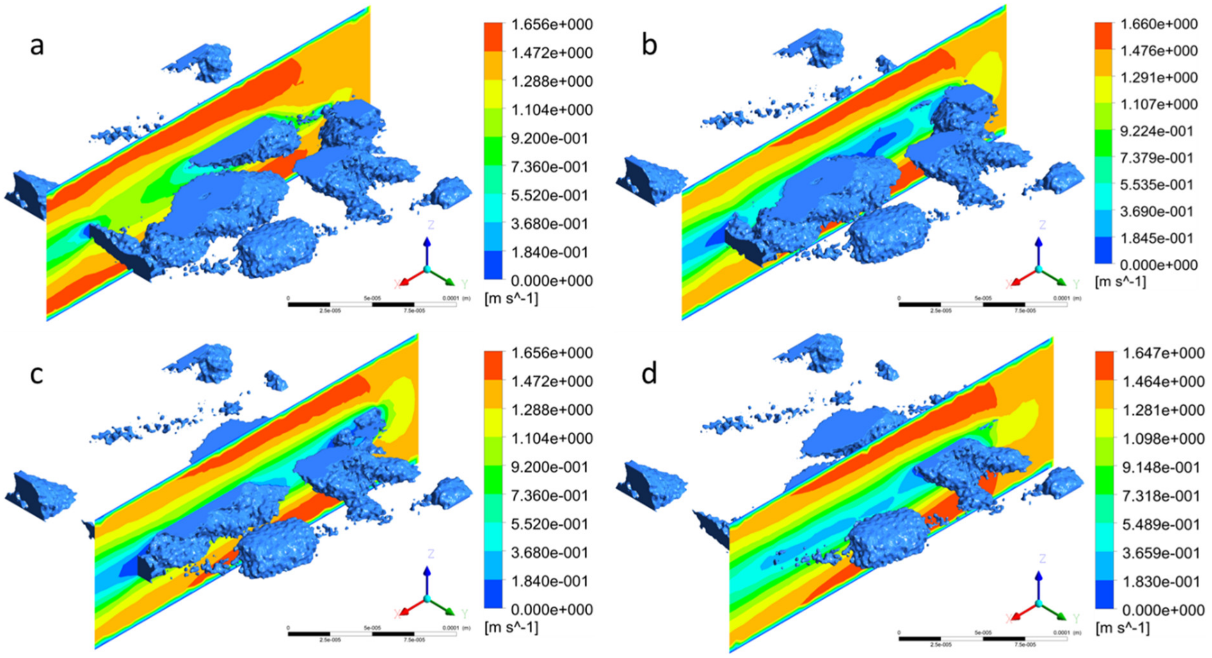

Supplementary Figure 43. (a-d) Velocity contour plots at the ZX plane for fluidic behavior at the **X** direction; Y height (+Y from the basal plane) for a-d are 230, 250, 270 and 290 µm, respectively.

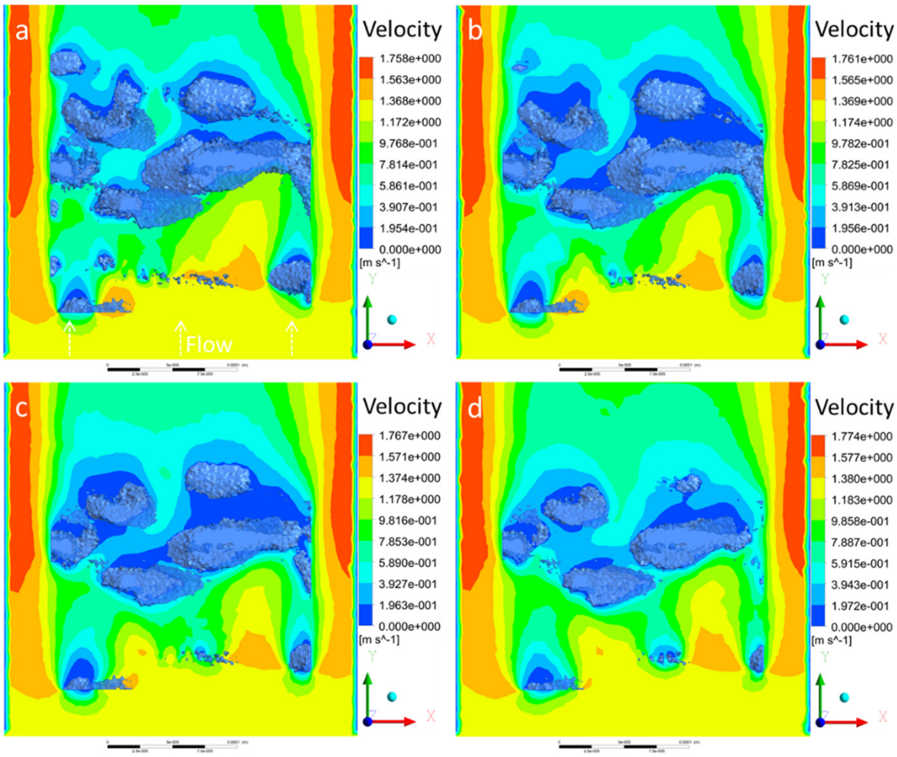

Supplementary Figure 44. (a-d) Velocity contour plots at the XY plane for fluidic behavior at the **Y** direction; Z height (+Z from the basal plane) for a-d are 40, 45, 50 and 55 μm, respectively.

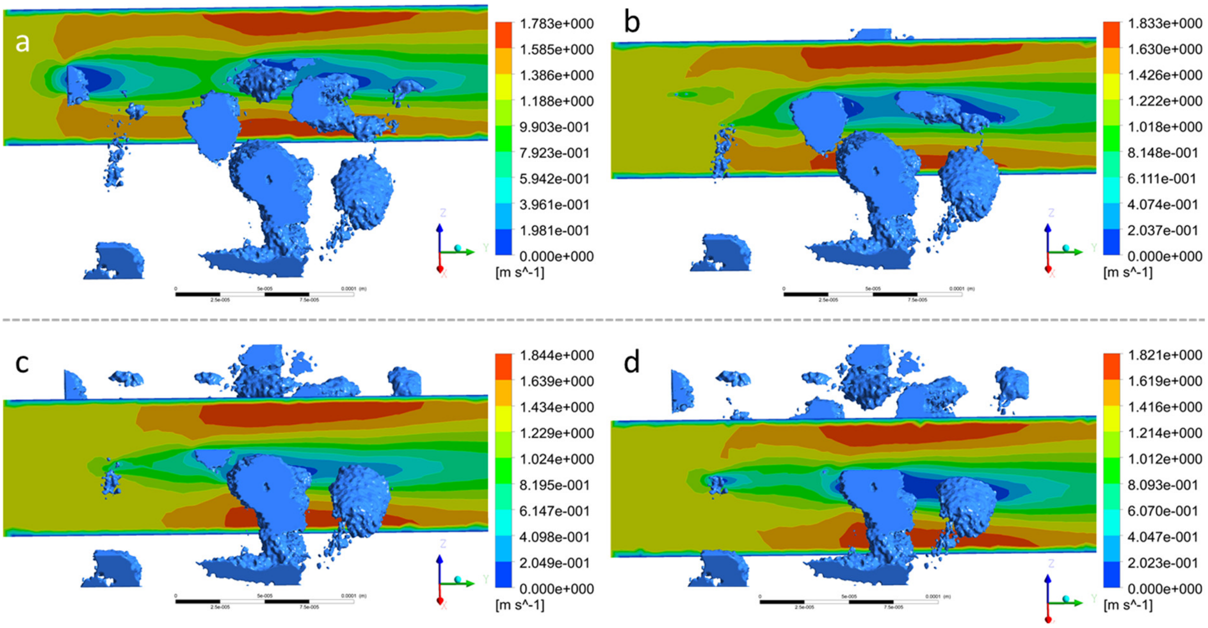

Supplementary Figure 45. (a-d) Velocity contour plots at the YZ plane for fluidic behavior at the **Y** direction; X height (+Y from the basal plane) for a-d are 120, 160, 200 and 240 μm, respectively.

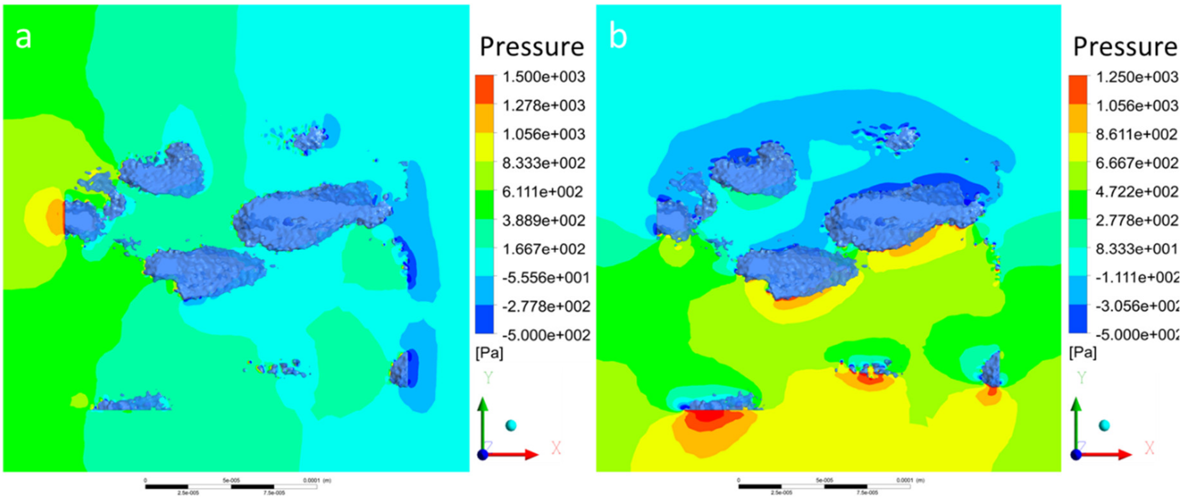

Supplementary Figure 46. Pressure contour plots at the XY plane for fluidic behavior at the (a) **X** and (b) **Y** direction; Z height (+Z from the basal plane) are 55 μm.

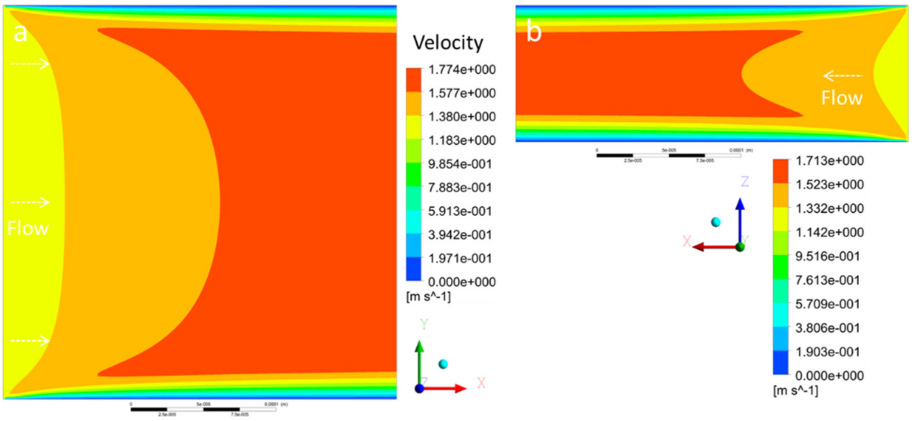

Supplementary Figure 47. Velocity contour plots for the blank model (without M_1-MoS_2-GF in the same model size) at the (a) XY and (b) ZX planes for fluidic behavior at the **X** direction. Z height for a is 55 μm. Y height for b is 130 μm.

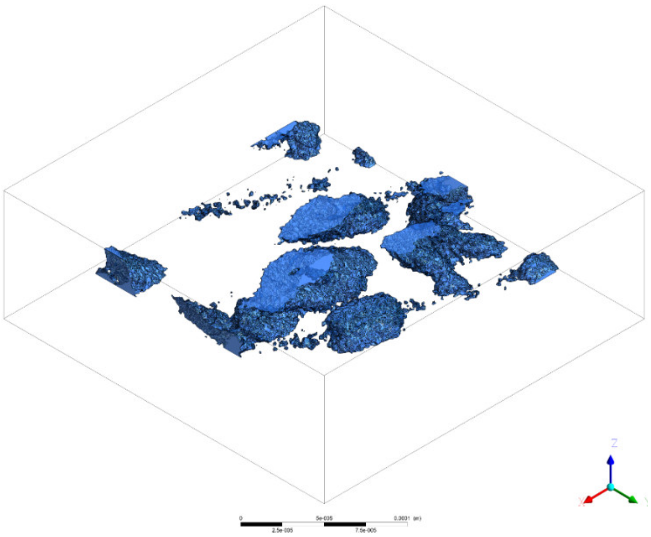

Supplementary Figure 48. 3D model for CFD calculations. The model was subtracted from the original structure from micro-CT and repaired in the CFD software.

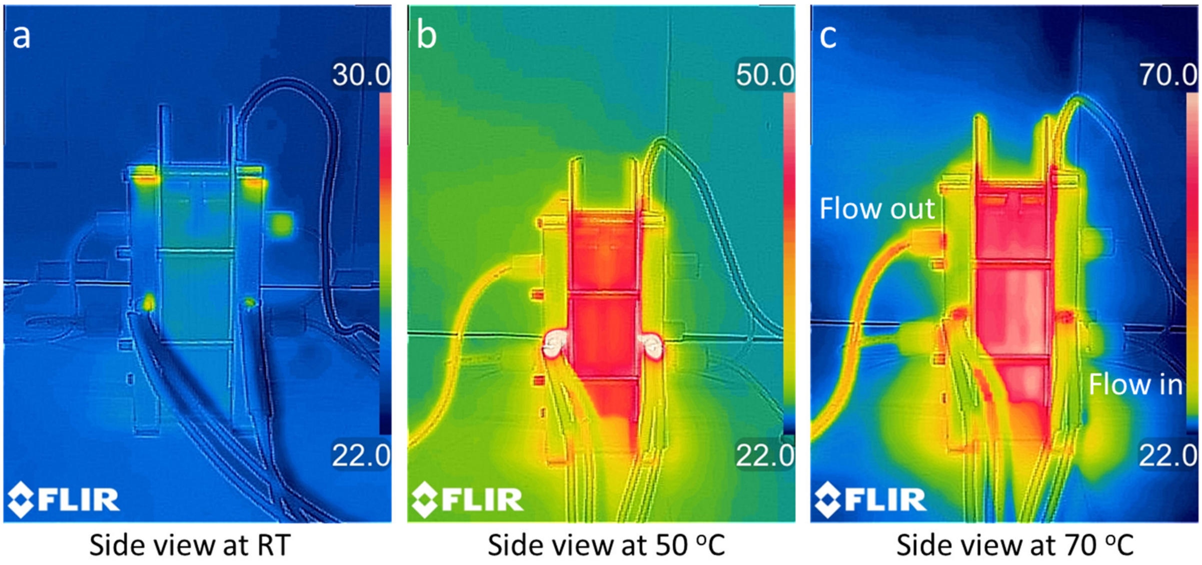

Supplementary Figure 49. (a) Thermal imaging (side view) of the flow cell operating at r.t.; (b) 50 °C and (c) 70 °C. The temperature gradient was uniform at the catalytic component while a hot flow-out solution was also detected (~ 64 °C at an operating temperature of 70 °C).

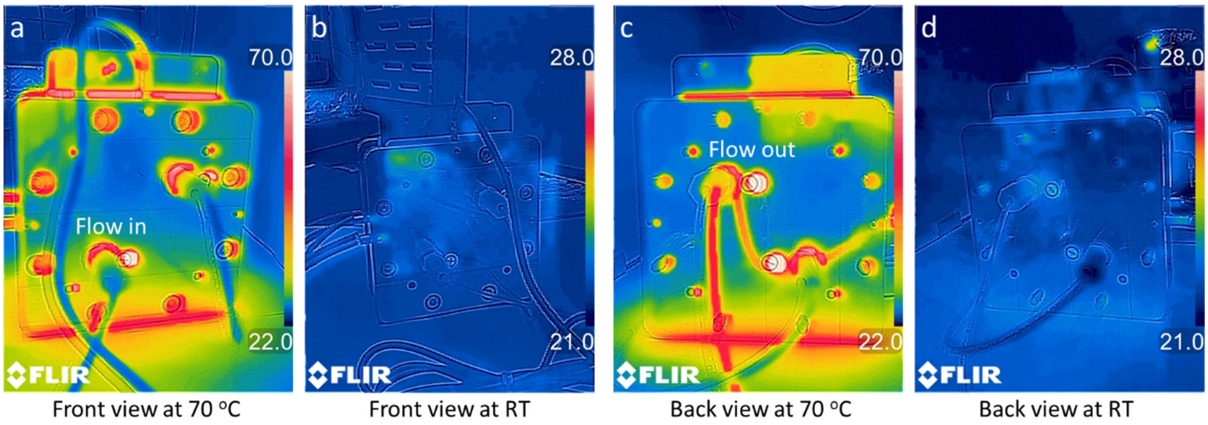

Supplementary Figure 50. (a) Thermal imaging (front view) of the flow cell operating at 70 °C and (b) r.t.; (c) Back view at 70 °C and (d) back view at r.t.

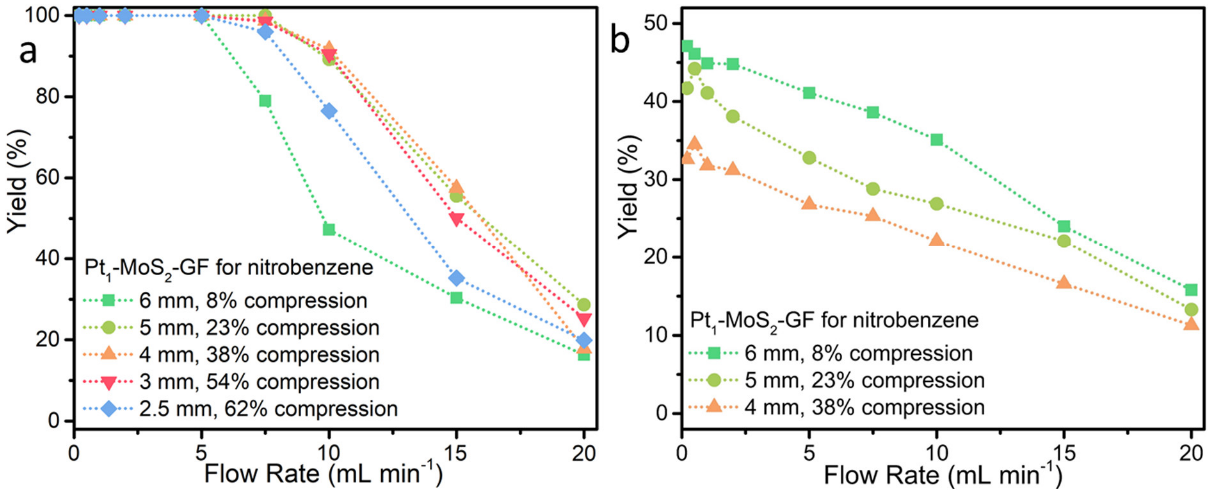

Supplementary Figure 51. Catalytic performance of Pt_1-MoS_2-GF for nitrobenzene reduction at various compression rates in the (a) quantitative conversion regime and (b) reaction limited regime. Condition: (a) 0.025 M nitrobenzene, 0.050 M ammonia borane in acetonitrile/H_2O mixture (5:1, v/v) at a flow rate of 1 mL min^{-1}; (b) 0.10 M nitrobenzene, 0.05 M ammonia borane in acetonitrile/H_2O mixture (5:1, v/v) at a flow rate of 7.5 mL min^{-1}.

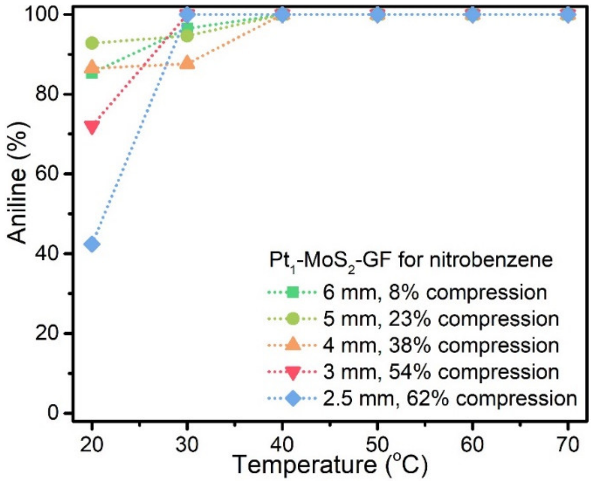

Supplementary Figure 52. Catalytic performance of Pt_1-MoS_2-GF for nitrobenzene reduction at various compression rates in the quantitative conversion regime. Condition: 0.025 M nitrobenzene, 0.050 M ammonia borane in acetonitrile/H_2O mixture (5:1, v/v) with 1 piece of Pt_1-MoS_2-GF (4 × 4 cm^2) at a flow rate of 1 mL min^{-1}.

Supplementary Tables

Supplementary Table 1. Results of the EXAFS fitting on PtO_2 and Pt_1-MoS_2.

	Bonding	N	R	Sigma^2	R-factor
PtO_2	Pt-O	6.0	2.01(0.02)	0.0012(0.0002)	0.003
Pt_1-MoS_2	Pt-S	3.7 (0.2)	2.318(0.004)	0.0019(0.0006)	0.0009

Supplementary Table 2. Correlation between Reynolds number (Re) and catalytic performance of Pt_1-MoS_2 for the reduction of nitrobenzene in the low-conversion regime (< 30%).

Flow Rate	Re (tubing)	Re (graphite plate)	Re (CFD model)	$TOF_{Pt1\text{-}MoS2}$
0.1 mL min^{-1}	2.4	4.7	20.2	18.5
2 mL min^{-1}	47	93	404	453.6
5 mL min^{-1}	117	234	1011	1161.2
10 mL min^{-1}	234	468	2022	1130.2

Reynolds number is calculated using the density (786 kg m^{-3}) and viscosity of acetonitrile (0.35 cP). Physical dimensions of plastic tubing, graphite plate and CFD model are provided in the experimental section. The Reynolds number may vary locally near the catalyst surface.

Supplementary Table 3. Correlation between the effectiveness factor (η) and the β value in the Weisz-Prater criterion for the given R_p (0.5 μm) and reaction order (n).

Reaction order (n)	effectiveness factor (η)	β value	Weisz-Prater criterion?
0	0.9	0.1	Yes ($\leq 3\beta$)
1	0.8	0.8	Yes ($\leq 3\beta$)
1	0.9	0.4	Yes ($\leq 3\beta$)
1	0.95	0.2	Yes ($\leq 3\beta$)
2	0.8	2.0	Yes ($\leq 3\beta$)
2	0.9	1.0	Yes ($\leq 3\beta$)
2	0.95	0.5	Yes ($\leq 3\beta$)

Supplementary Table 4. The Bader charge and *d*-band centre of Pt atom.

Model	Bader charge (e^-)	*d*-band centre (eV)
Pt-3S at the Mo atop	9.924	-2.689
Pt (111) surface	10.046	-2.451

Supplementary Table 5. List of bonding orbitals from -pCOHP calculations.

-pCOHP for Pt-S		-pCOHP for Pt-S with nitro		-pCOHP for Pt-O with nitro	
Pt - S Bonding	Area[1]	Pt - S Bonding	Area	Pt - O Bonding	Area
$6s - 3p_z$	0.36837	$5d_{yz} - 3p_y$	0.38119	$6s - 2p_z$	0.42563
$5d_{yz} - 3p_y$	0.3469	$6s - 3p_z$	0.25959	$5d_{z2} - 2p_x$	0.04821
$5d_{xy} - 3p_x$	0.19743	$5d_{xy} - 3p_x$	0.18051		
$5d_{x2-y2} - 3p_y$	0.16107	$6s - 3p_y$	0.16031		
$6s - 3p_y$	0.0978	$5d_{x2-y2} - 3p_y$	0.13446		

-pCOHP for Pt-Pt on Pt (111)		-pCOHP for Pt-Pt with nitro on Pt (111)		-pCOHP for Pt-O with nitro on Pt (111)	
Pt - Pt Bonding	Area	Pt - Pt Bonding	Area	Pt - O Bonding	Area
$6s - 6s$	0.188	$6p_y - 6s$	0.193	$5d_{z2} - 2p_x$	0.056
$5d_{xy} - 6s$	0.044	$6s - 6p_y$	0.175	$6s - 2p_z$	0.038
$6s - 5d_{xy}$	0.041	$6p_y - 6p_x$	0.132	$6s - 2p_x$	0.034
		$6p_x - 6p_y$	0.123		
		$6p_y - 5d_{xy}$	0.104		
		$5d_{xy} - 6p_y$	0.103		
		$6p_x - 5d_{x2-y2}$	0.090		
		$5d_{x2-y2} - 6p_x$	0.088		
		$6s - 6s$	0.071		

[1]Integrated area of the -pCOHP of the valance band ($E_F \leq 0$). For Pt-3S and nitro on Pt-3S, the Pt metal center adopts a [*d3s*] orbital hybridisation involving the $6s$, $5d_{yz}$, $5d_{xy}$ and $5d_{x2-y2}$ to form a tetrahedral complex.

Supplementary References

[1] Zhang, B., Liu, J., Wang, J., Ruan, Y., Ji, X., Xu, K., Chen, C., Wan, H., Miao, L. & Jiang, J. Interface engineering the $Ni(OH)_2/MoS_2$ heterostructure for highly efficient alkaline hydrogen evolution. *Nano Energy* **37**, 74–80 (2017).

[2] Zhang, H., Yu, L., Chen, T., Zhou, W. & Lou, X. W. D. Surface modulation of hierarchical MoS_2 nanosheets by Ni single atoms for enhanced electrocatalytic hydrogen evolution *Adv. Funct. Mater.* **28**, 1807086 (2018).

[3] Du, Y., Zhu, Y., Xi, S., Yang, P., Moser, H. O., Breese, M. B. H. & Borgna, A. XAFCA: a new XAFS beamline for catalysis research. *J. Synchrotron Rad.* **22**, 839–843 (2015).

[4] Ravel, B. & Newville, M. ATHENA, ARTEMIS, HEPHAESTUS: data analysis for X-ray absorption spectroscopy using IFEFFIT. *J. Synchrotron Rad.* **12**, 537–841 (2005).

[5] Emmel, D., Hofmann, J. D., Arlt, T., Manke, I., Wehinger, G. D. & Schröder, D. Understanding the impact of compression on the active area of carbon felt electrodes for redox flow batteries. *ACS Appl. Energy Mater.* **3**, 4384–4393 (2020).

[6] Kresse, G. & Hafner, J. *Ab initio* molecular dynamics for liquid metals. *Phys. Rev. B* **47**, 558–561 (1993).

[7] Kresse, G. & Furthmuller, J. Efficient iterative schemes for *ab initio* total-energy calculations using a plane-wave basis set. *Phys. Rev. B* **54**, 11169–11186 (1996).

[8] Perdew, J. P., Burke, E. & Ernzerhof, M. Generalized gradient approximation made simple. *Phys. Rev. Lett.* **77**, 3865–3868 (1996).

[9] Blöchl, P. E. Projector augmented-wave method. *Phys. Rev. B* **50**, 17953–17979 (1994).

[10] Dronskowski, R. & Bloechl, P. E. Crystal orbital Hamilton populations (COHP): energy-resolved visualization of chemical bonding in solids based on density-functional calculations. *J. Phys. Chem.* **97**, 8617–8624 (1993).

[11] Deringer, V. L., Tchougréeff, A. L. & Dronskowski, R. Crystal orbital Hamilton population (COHP) analysis as projected from plane-wave basis sets. *J. Phys. Chem. A* **115**, 5461–5466 (2011).
